# Creating Ferroelectricity and Ultrahigh-Density Polar Skyrmion in Paraelectric Perovskite Oxide Monolayers by Moiré Engineering

**DOI:** 10.34133/research.0621

**Published:** 2025-02-17

**Authors:** Tao Xu, Tao Qian, Jiafei Pang, Jingtong Zhang, Sheng Li, Ri He, Jie Wang, Takahiro Shimada

**Affiliations:** ^1^ Department of Mechanical Engineering and Science, Kyoto University, Nishikyo-ku, Kyoto 615-8540, Japan.; ^2^ Ningbo Institute of Materials Technology and Engineering, Chinese Academy of Sciences, Ningbo 315201, China.; ^3^ Department of Engineering Mechanics, School of Aeronautics and Astronautics, Zhejiang University, Hangzhou 310027, China.; ^4^ Department of Civil and Earth Resources Engineering, Kyoto University, Nishikyo-ku, Kyoto 615-8540, Japan.

## Abstract

Atomic-scale polar topologies such as skyrmions offer important potential as technological paradigms for future electronic devices. Despite recent advances in the exploration of topological domains in complicated perovskite oxide superlattices, these exotic ferroic orders are unavoidably disrupted at the atomic scale due to intrinsic size effects. Here, based on first-principles calculations, we propose a new strategy to design robust ferroelectricity in atomically thin films by properly twisting 2 monolayers of centrosymmetric SrTiO_3_. Surprisingly, the emerged polarization vectors curl in the plane, forming a polar skyrmion lattice with each skyrmion as small as 1 nm, representing the highest polar skyrmion density to date. The emergent ferroelectricity originates from strong interlayer coupling effects and the resulting unique strain fields with obvious ion displacements, contributing to electric polarization comparable to that of PbTiO_3_. Moreover, we observe ultraflat bands (band width of less than 5 meV) at the valence band edge across a wide range of twist angles, which show widths that are smaller than those of common twisted bilayers of 2-dimensional materials. The present study not only overcomes the critical size limitation for ferroelectricity but also reveals a novel approach for achieving atomic-scale polar topologies, with important potential for applications in skyrmion-based ultrahigh-density memory technologies.

## Introduction

Topological entities exhibiting skyrmionic textures have been a topic of intense research due to their rich emerging physical phenomena and potential for enabling innovative applications, such as racetrack memories [1,2]. A variety of exotic polar topological domains, such as the flux-closure domain [3], polar vortex [4–7], polar skyrmion [8,9], polar meron [10,11], and periodic polar wave [12], have gradually been discovered in ferroelectric nanostructures and superlattices in recent years. The creation of these ferroelectric topological textures relies strongly on the geometrically induced extrinsic chiral interactions arising from the delicate interplay among ferroelectric ordering, electrostatics, strain, and related gradients. With the rapid increase in data volume, minimizing the size of these topological domains is crucial for enhancing the speed and capacity of integrated devices in future ultrahigh-density data storage devices. However, because of the robust coupling between the lattice and polarization in ferroelectrics, polarization rotation in these topological structures results in considerable energy expenditure, involving substantial elastic and polarization gradient energies. These energy costs increase markedly with decreasing size of polar skyrmions. Consequently, creating ultrasmall skyrmions is challenging, typically with their sizes exceeding 10 nm [8] in ferroelectric systems. Furthermore, it is also widely recognized that ferroelectricity completely disappears below a critical thickness of several nanometers in ferroelectrics due to the effect of the depolarization field [13,14], although the precise value of the critical thickness is affected by mechanical and electrical conditions. Therefore, devising an innovative approach that transcends conventional geometric confinement is highly desirable and timely for advancing the miniaturization of polar skyrmions and systems that accommodate them.

Strain engineering through heteroepitaxy is a promising technique for the modulation of various structural [15,16], ferroelectric [17,18], and electric properties [19] of perovskite oxides due to the intrinsic pronounced electromechanical coupling effect. Furthermore, recent research has innovatively proposed the use of strain gradients alone in nanofilms with different mechanical wrinkles, such as a bulge and checkboard morphology, to create diverse polar topologies and phase transitions [20]. Nevertheless, these mechanical deformations largely depend on the elastic properties of the material and structural dimensions, and precise control of the required regular deformation patterns has been elusive in practice. On the other hand, the moiré superlattices formed by various twisted bilayers have been created, becoming versatile platforms for exploring a myriad of intriguing properties [21–23], in which lattice distortions and local strain play the key role. In addition to 2-dimensional (2D) atomic layers, artificial twisted stacks of freestanding perovskite oxide thin films with arbitrary twist angles have also been fabricated recently [24–26], opening up the possibility of exploring conventionally unavailable moiré-related strain patterns and associated exotic domain configurations. However, due to the experimental challenges in directly mapping the reconstruction mechanics and characterizing the displacement fields of these moiré materials, the discovery of novel emergent phenomena and exotic domain configurations in these twisted perovskite oxides remains largely unexplored.

In this study, we perform atomic simulations of twisted perovskite oxide bilayers to investigate the lattice reconstruction, translational symmetry breaking, and consequential ferroelectric properties in SrTiO_3_ moiré superlattices. Our findings reveal that pronounced interfacial charge transfer and the resulting large structural reconstruction, featuring unique displacement patterns that are difficult to observe in traditional experiments, contribute to atomic-thin ferroelectricity characterized by polar skyrmions. We also obtain ultra-flat bands at the valence band edge in the SrTiO_3_ moiré superlattices. Our results demonstrate that “moiré engineering” offers an unprecedented platform for overcoming the critical thickness limitation and creating atomic-scale ferroelectricity characterized by exotic polar topologies in perovskite oxides.

## Method

All first-principles calculations were carried out within the density functional theory (DFT) framework using the VASP code [27,28]. The electronic wave function was expanded in a plane-wave basis set, with a plane-wave cutoff energy set to 500 eV. We utilized the projector-augmented wave (PAW) pseudopotential method [29], treating the valence electrons as follows: the 4*s*, 4*p*, and 5*s* electrons for Sr; the 3*s*, 3*p*, 3*d*, and 4*s* electrons for Ti; and the 2*s* and 2*p* electrons for O. The exchange-correlation energy was handled using the generalized gradient approximation (GGA) with the Perdew–Burke–Ernzerhof (PBE) functional [30]. The Brillouin zone for both bulk and bilayer SrTiO_3_ was sampled using a Monkhorst–Pack *k*-point mesh of 8 × 8 × 1, whereas for moiré superlattices, a 3 × 3 × 1 *k*-point mesh was employed. A large vacuum space (>15 Å) normal to the layer plane was applied to the bilayer and moiré superlattices to avoid spurious interactions. Relaxations of the atomic positions were iterated until both the energy change per unit cell and the Hellmann–Feynman force on each atom were below the thresholds of 10^−6^ eV and 0.01 eV Å^−1^, respectively.

## Results and Discussion

**Fig. 1.**
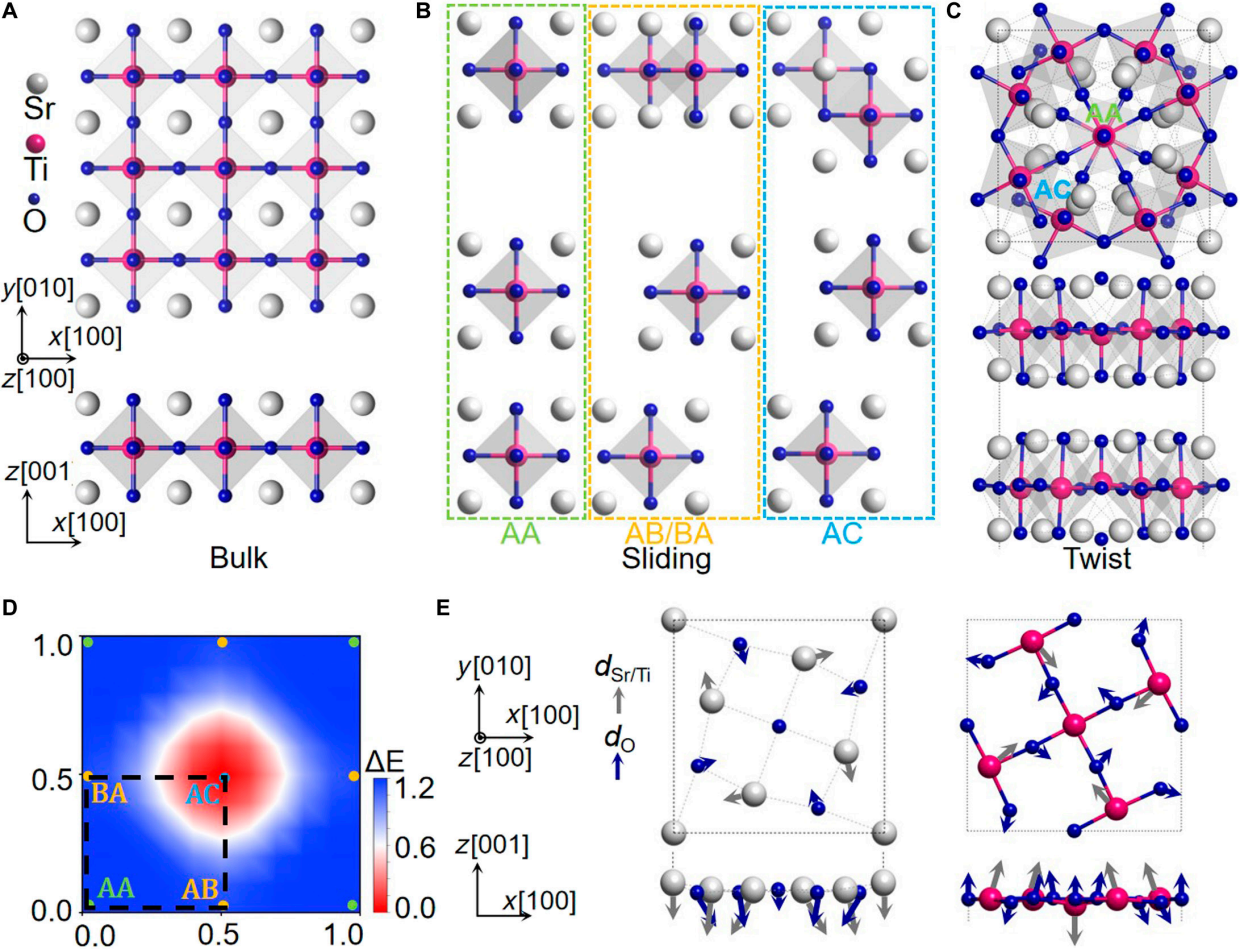
Simulation model. (A) Bulk SrTiO_3_ structure. (B) Different stackings for SrTiO_3_ bilayer. (C) Moiré unit cell with *θ* = 36.87°. (D) Energy landscape for the bilayer sliding in the *xy* plane. (E) Atomic displacements in the upper layer of moiré unit cell.

SrTiO_3_ is a benchmark oxide perovskite with a cubic structure and a calculated lattice parameter of *a*_c_ = 3.89 Å (Fig. 1A). Inspired by recent experimental progress in the fabrication of freestanding SrTiO_3_ thin films down to the monolayer limit [31] and twisted stacks of SrTiO_3_ nanomembranes [25,26], here we theoretically investigate twisted bilayer SrTiO_3_ with the thickness of each layer *N* ranging from 1 to 6 formula units (f.u.). The initial configurations of thin films with cubic lattice coordinates are cleaved along the (001) crystallographic plane from the bulk SrTiO_3_. There are 2 different terminations for the (001) surfaces of the films, i.e., the SrO and TiO_2_ terminations. As a result, bilayer structures with 3 different terminations can be constructed, with their relative stabilities discussed in detail in the Supplementary Materials. Our preliminary DFT computational assessments indicate that TiO_2_–TiO_2_ termination forms complicated chemical bonds between the interfaces after relaxation (Fig. S1), whereas the SrO–SrO interface results in van der Waals-type interlayer interactions with an interlayer spacing of approximately 3.32 Å. The fabrication of SrO–SrO interfacial structures has also been experimentally realized [32,33]. Therefore, we focused on the stacking of SrTiO_3_ thin films with SrO–SrO interface. Figure 1B illustrates 3 high-symmetry stacking configurations in this bilayer SrTiO_3_: In the AA stacking arrangement, the upper and lower layers are perfectly aligned. The AB/BA stacking pattern results from shifting the upper layer by (0.5*a*, 0) or (0, 0.5*a*) along the *x* and *y* directions, respectively. The AC stacking corresponds to moving the upper layer by (0.5*a,* 0.5*a*), known as the well-studied Ruddlesden–Popper interface [32–35]. The stacking energy of the SrTiO_3_ bilayer is calculated as the upper layer slides with respect to the lower layer, and the results are depicted in Fig. 1D. It is evident that the AC stacking exhibits the lowest energy, whereas the AB/BA stacking and AA stacking configurations have higher energy due to remarkable repulsion between the same elements across the interfaces.

We follow previously reported procedures [34] to construct square-shaped twisted supercell structures with commensurate twist angles. Given the ability to fabricate twisted structures with arbitrary twist angles in experiments and considering the availability of computational resources, we specifically focus on large-angle models (i.e., small supercells) with twisting angles *θ* > 10° in the present study, as listed in Table S1. For the moiré structure with the largest twist angle of 36.87°, we investigate the thickness-dependent properties by building a series of STO*_N_*/STO*_N_* (*N* = 1 to 6 unit cells) superlattices, where *N* indicates the thickness of each sublayer. The unit cells of the moiré superlattice (*N* = 1) with the twist angles of *θ* = 36.87° and 22.62° are illustrated in Fig. 1C and Fig. S2, which exhibit moiré periodicity λ values of 8.7 and 14.0 Å, respectively. The rigidly twisted SrTiO_3_ thin films are then relaxed utilizing DFT simulations to capture their intralayer and interlayer interactions.

**Fig. 2.**
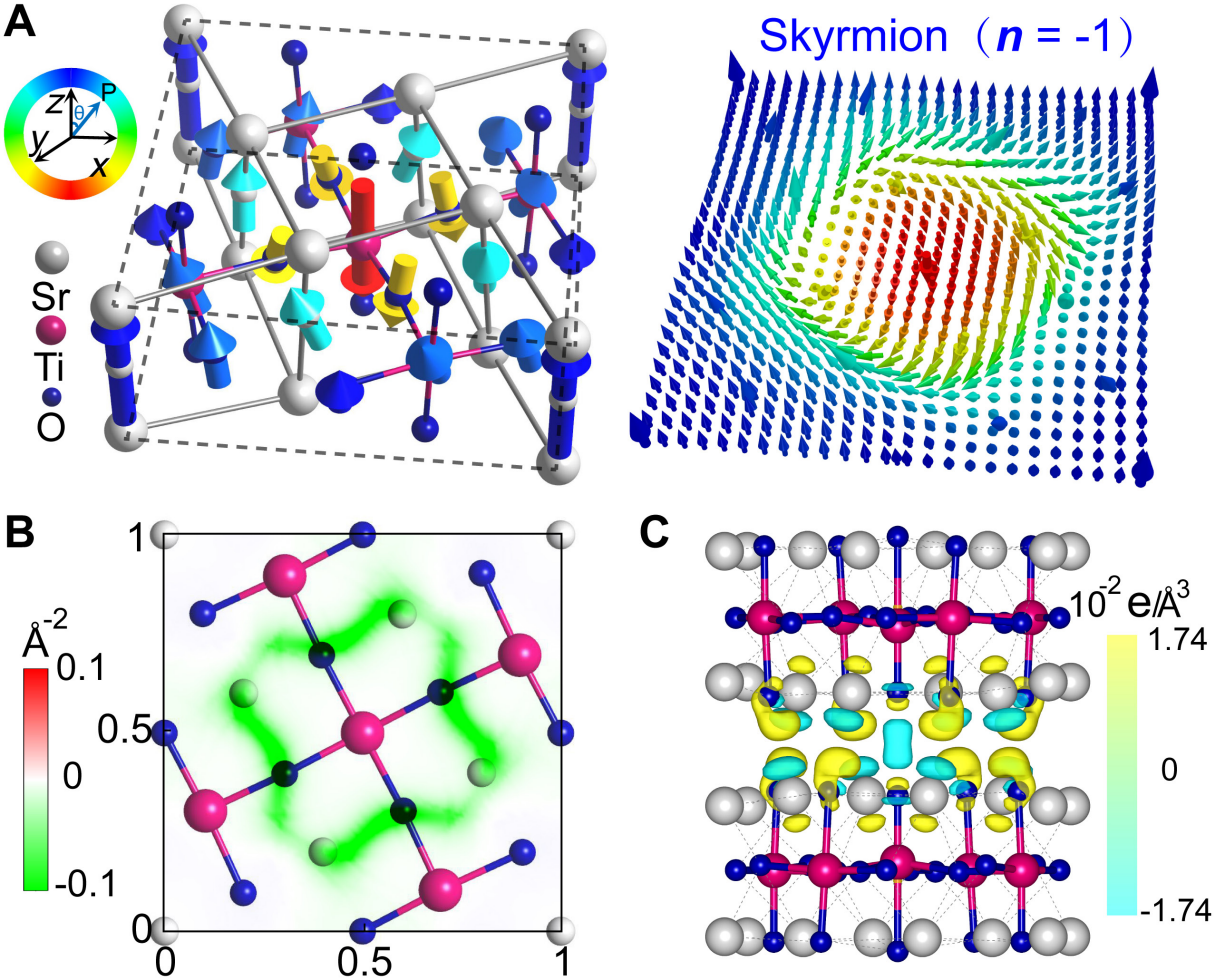
(A) Polarization distribution in the upper layer of moiré unit cell with *θ* = 36.87°. Color denotes the angle from the *z* direction. The right panel shows an interpolated and normalized vector field of local polarization. (B) Distribution of topological charge density. (C) Difference charge density plots. The isosurfaces are 2.5 × 10^−3^ e/bohr^3^.

We begin by investigating the moiré superlattice with *N* = 1 and *θ* = 36.87°. As illustrated in Fig. 1E, the twisted SrTiO_3_ bilayers after full relaxation exhibit considerable local lattice distortions and atomic displacements relative to the thin-film structure. The center and boundary atoms exhibit pure out-of-plane displacements. The atoms in the transition zone exhibit a complex 3D displacement configuration, which is primarily composed of in-plane torsional displacements and unequal out-of-plane displacements. These notable off-centered displacements cause the centers of positive and negative charges in the local unit cell to deviate from the original centrosymmetric structure, giving rise to local electric polarization in the otherwise paraelectric SrTiO_3_ ultrathin films. The polarization properties of the twisted layers are investigated by using the site-specific local electric polarization *p_i_* (Note S3), calculated using the Born effective charge tensors as $p_{i}=\frac{e}{\Omega_{c}}\;\omega_{j}Z_{j}u_{j}$ [36,37]. Note that the local polarizations are defined continuously on different atoms by using atom-centered unit cells, as illustrated in Fig. S16. The local polarization, defined based on different choices of unit cells, is illustrated in Fig. S17A to C, showing sharp variations at the atomic scale. These large variations arise from the considerable differences in charge redistribution within a unit-cell region and at the atomic scale (see Fig. 2C), which will be discussed latter. To clearly capture the dramatic spatial variation of the local polarization without losing the polarization information due to the different choices of unit cells, we integrate the obtained local polarization from different unit cell-based dipoles and present them in the same image (Fig. S17D). This approach enables a more accurate capture of spatial variations in local polarization at the atomic scale. Similar subunit-cell-level local polarization information has also been widely used in the investigation of polar topological structures, both experimentally and theoretically [38–42]. The obtained 3D ferroelectric polarization patterns on the upper layer are shown by bold arrows in Fig. 2A, with the length and color reflecting the magnitude and direction of polarization, respectively. For clarity, the in-plane and out-of-plane directions are also shown separately in Fig. S3, which depicts an in-plane vortex-like polarization and an out-of-plane antiparallel polarization. The emergence of these local polarizations in SrTiO_3_ indicates that we have indeed developed ferroelectricity in nonferroelectric materials with atomic-scale thickness. It is important to note that this is not feasible even in intrinsically ferroelectric perovskite oxides, such as PbTiO_3_ and BaTiO_3_, because of the constraints imposed by the critical size. More interestingly, the local electric dipoles gradually transition from a downward orientation to an upward orientation as they extend from the core to the periphery. The polarization distribution on the lower layer exhibits inverse complex textures. These unique outward polarization vector arrangements, characterized by the radial reversal of the out-of-plane component from the center to the periphery, are reminiscent of the quasi-2D topological order known as a skyrmion.

The mathematical characterization of the topological nature of the polarization pattern can be evaluated through the introduction of the topological charge *n* defined as

$$n=\iint \frac{1}{4\pi }u\cdot \left(\frac{\partial u}{\partial x}\times \frac{\partial u}{\partial y}\right)\text{dxdy},$$ (1)

where ***u*** is a normalized electric polarization vector continuously defined across the entire integrated plane. Given that the current atomic-scale ferroelectric polarization caused by “moiré engineering” is restricted to such a small area and lacks perfect spatial continuity, the direct application of this definition may lead to inaccuracies. Hence, the discrete polarization vector field in the moiré superlattice is interpolated to assess the topological charge. As illustrated in the right panel of Fig. 2A, the resulting interpolated local electric polarization, represented by thin arrows, aligns with the original discrete polarization (bold arrows) and preserves the topological characteristics. The polar configuration clearly exhibits a hedgehog-like structure, with the polarization direction (indicated by the colors) smoothly rotating from downward to upward and from the center to the edge of the moiré superlattice. The resulting topological number is approximately –1.0 (with a value of –1.06 calculated without interpolated local electric polarization), confirming that the polarization pattern in the SrTiO_3_ moiré superlattice is truly an electric equivalent of a magnetic skyrmion, with dimensions smaller than 1 nm. The corresponding topological charge density distribution is also plotted in Fig. 2B and shows topological charge density accumulation primarily at the periphery of the skyrmion, resulting in a star-like shape with 4 rounded arms. We also investigated the effect of PBESol pseudopotentials on the polarization distribution and found no pronounced change in the emerging polar skyrmion patterns (see Fig. S4). Notably, the lower layer also has a skyrmion configuration with a topological number of 1.0, in which the out-of-plane polarizations are opposite to those in the upper layer. The whole polar configuration (see Fig. S5) is analogous to that of a pair of antiferromagnetic-coupled magnetic skyrmions observed in magnetics [43] and can be referred to as polar bilayer-skyrmion. Moreover, both skyrmions exhibit right-handed chirality. In contrast, when the twist occurs in the opposite direction with *θ* = –36.87°, a polar bilayer-skyrmion still forms but with left-handed chirality (see Fig. S6). This observation indicates that the direction of the twisting angle can influence the chirality of the resulting skyrmion.

Previous studies have suggested that specific stacking of nonferroelectric van der Waals layers with honeycomb lattices may break symmetry and induce vertical polarization, known as sliding ferroelectricity. However, a recent general theory of bilayer stacking ferroelectricity indicates that sliding ferroelectricity is not permitted when 2 monolayers with the *D*_4*h*_ point group (e.g., SrTiO_3_) are stacked [44]. Our first-principles calculations indeed confirm that bilayer SrTiO_3_ exhibits negligible sliding-induced ferroelectricity across all stacking configurations, as illustrated in Fig. 1B. Instead, the underlying mechanism for the formation of twisting-induced skyrmion, characterized by an in-plane vortex-like polarization and an out-of-plane polarized core, is attributed to the varying energetics of different stacking domains and robust interaction between the 2 layers. In the central region of the moiré superlattice, where the local atomic structure resembles the high-energy AA stacking domains, the O atoms of the 2 layers align vertically, resulting in strong repulsion between them. These 2 O atoms strongly repel each other, i.e., the center O atom in the upper layer shifts upward, while the other in the lower layer shifts downward, giving rise to downward and upward polarization, respectively, for the central region of the upper and lower layers. By contrast, in the middle transition regions of the AC stacking domain, the interfacial O anions (or Sr cations) in the upper layer are located approximately above the Sr cations (or O anions) in the lower layer and will tend to create Sr–O bonds. The bonding characteristics induced by the interface interaction can be discerned from the charge density difference as illustrated in Fig. 2C, in which oxygen anions gain electrons and Sr cations lose electrons. The downward movement of oxygen atoms in the upper layer breaks the out-of-plane symmetry and contributes to the upward polarization in the AC stacking region of the upper layer. As a result, the out-of-plane polarization component in the upper layer points downward at the center, gradually decreases, and then changes to the outward direction. The strong interfacial attraction of O anions and Sr cations also induces a torsion-like displacement field, as illustrated in Fig. 1E, contributing to the in-plane vortex polarization. This in-plane vortex pattern is superimposed on the antiparallel out-of-plane polarization, forming a polar skyrmion in SrTiO_3_ with a single unit cell thickness.

**Fig. 3.**
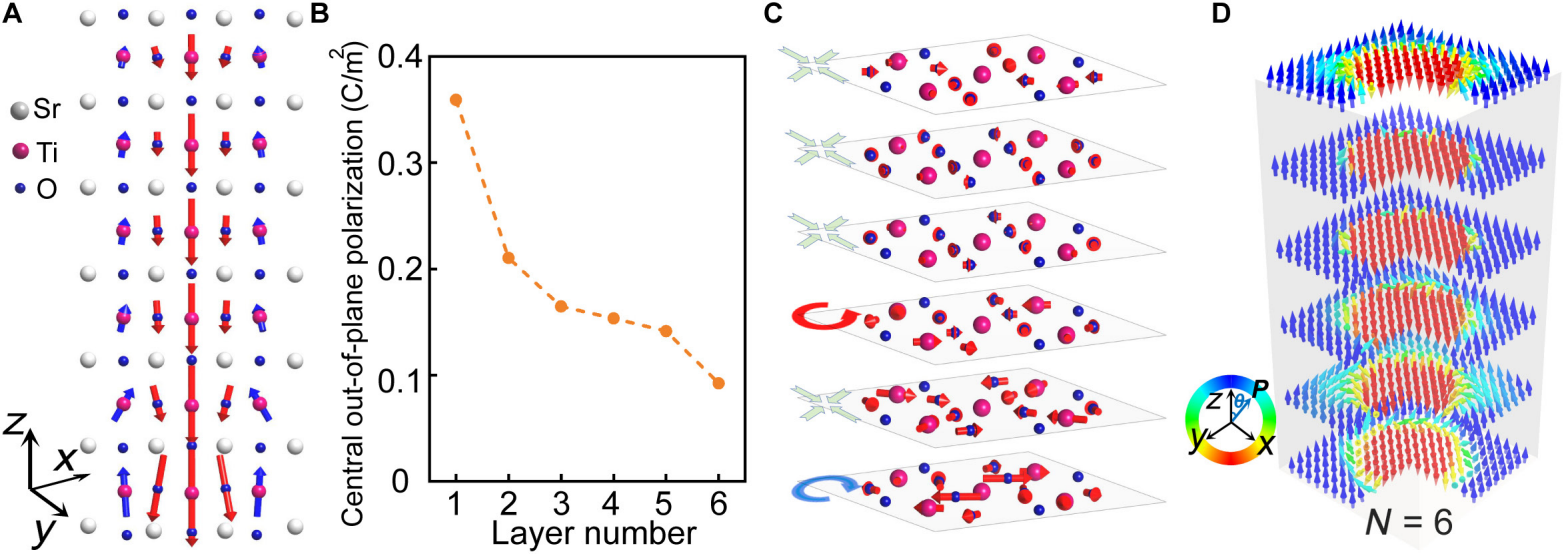
Ferroelectric properties in twisted SrTiO_3_ bilayer at *θ* = 36.87° and *N* = 6. (A) Out-of-plane polarization distribution in each TiO_2_-centered unit cell of the upper layer. (B) Central out-of-plane polarization in each unit cell of the upper layer. (C) In-plane polarization distribution in each TiO_2_-centered unit cell of the upper layer. (D) Interpolated and normalized vector field of local polarization in each unit cell of the upper layer.

Furthermore, we investigated the effects of increased bilayer thickness on the ferroelectricity and stability of polar topological structures in twisted bilayers. For a thickness of *N* = 2 f.u., the local polarization of the center unit cell in the interfacial sublayer decreases from 0.68 C/m^2^ at *N* = 1 to 0.44 C/m^2^. As the thickness further increases, the local polarization magnitude continues to decrease (see Fig. S7) but remains comparable to that observed in barium titanate [45]. Emerging ferroelectricity and polar skyrmions also persist in twisted structures at all thicknesses investigated. As illustrated in Fig. 3A, for the layer-by-layer polarization in the twisted bilayer with *N* = 6, the out-of-plane polarization points downward at the center, with antiparallel polarization and polarization rotations at the periphery; this pattern is consistent across all sublayers. The central local polarization in layer1 (the interfacial layer) exhibits the highest value due to strong interfacial effects, while the polarization values decrease sharply in the subsequent upper layers (Fig. 3B). Moreover, the in-plane polarization has either a vortex or center-convergent configuration (Fig. 3C) arising from the complex relative displacements of cations and anions (Fig. S8) due to both the inter- and intralayer effects. The superposition of in-plane and out-of-plane polarizations forms the skyrmions in all the sublayers (Fig. 3D). Since twisted bilayer SrTiO₃ films have already been successfully fabricated in experiments [25,26], polar skyrmions could potentially be observed at the proposed twisting angle using advanced techniques such as 4D scanning transmission electron microscopy diffraction imaging [42] and multislice electron ptychography [46]. On the other hand, the twisting angles also influence the resulting polarization pattern in the otherwise nonferroelectric SrTiO_3_. For the SrTiO_3_ moiré bilayers with a twisting angle of 22.62°, the polar skyrmions with a topological charge of –1 also appear, as illustrated in Fig. S2B. However, at smaller twist angles with larger supercells, while ferroelectricity continues to manifest, the skyrmion is supplanted by a more complex 3D polarization configuration, characterized by a combination of multiple in-plane clockwise vortices and out-of-plane antiparallel polarization (see Fig. S9).

**Fig. 4.**
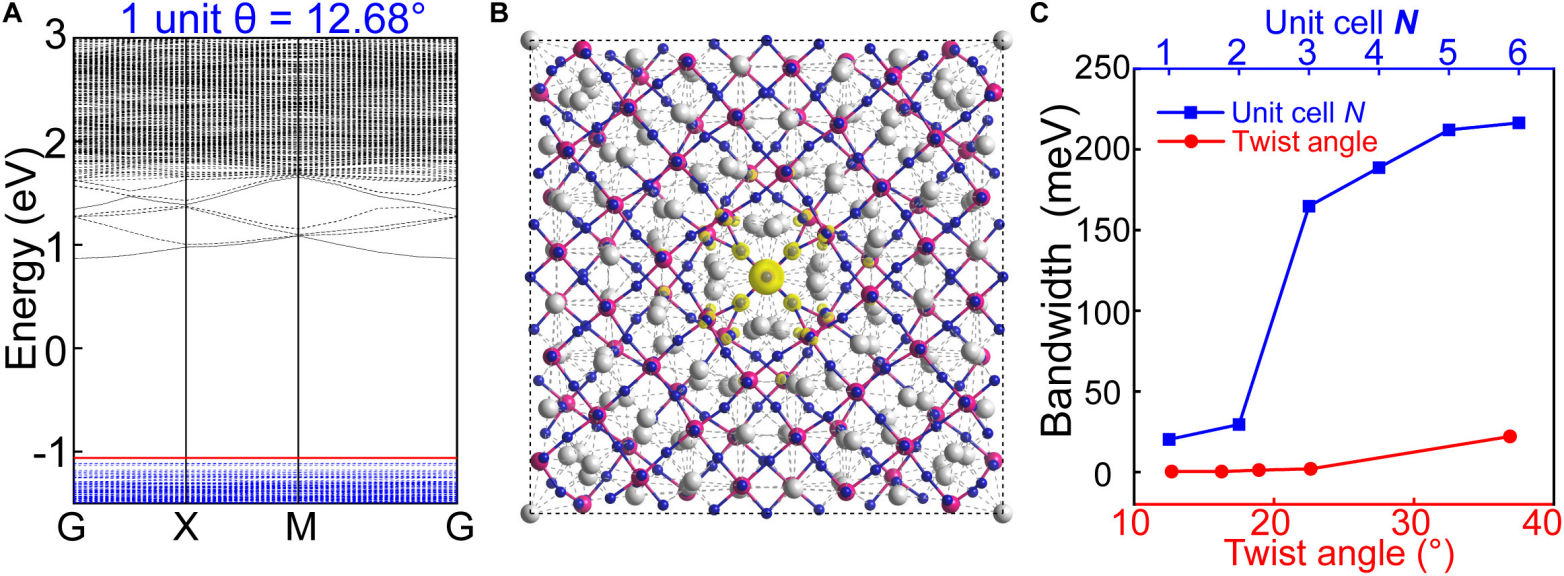
Electronic properties of twisted SrTiO_3_ bilayer. (A) Band structure of twisted SrTiO_3_ bilayer with twist angles of 12.68° and *N* = 1. (B) Charge density of the valence band maximum, in which the isosurfaces are 2 × 10^−3^ e/bohr^3^. (C) Thickness- and twist-angle-dependent bandwidth of the valence band maximum at *θ* = 36.87° and *N* = 1, respectively.

In addition to stabilizing polar topological structures, twisting engineering simultaneously induces noticeable variations in the electronic structure of SrTiO_3_, as depicted in Figs. S10 and S11 and Fig. 4. For bulk SrTiO_3_, both the conduction and valence bands exhibit notable dispersion (see Fig. S12). Upon twisting the bilayer at an angle of 36.87°, the top of the valence band tends to become flat. Its bandwidth decreases substantially with decreasing thickness *N* of the twisted bilayers (see the blue line in Fig. 4C) and reaches as low as 22.13 meV for *N* = 1. Furthermore, different twist angles also strongly affect the bandwidth. As shown by the red line in Fig. 4C (with the corresponding band structures illustrated in Fig. S10), for the twisted SrTiO₃ bilayer with a thickness of *N* = 1, the bandwidth decreases as the twist angle decreases within the range of large angles investigated. It is interesting that flat bands less than 5 meV appear for a wide range of these large twist angles. The minimum value, with an exceptionally narrow bandwidth of 0.24 meV along the high-symmetry lines of the first Brillouin zone (see Fig. 4A), is observed at a twist angle of 12.68°. The emergence of flat bands has drawn considerable research attention because of their link to strong-coupling superconductivity and correlated electron behavior [47,48]. The present result is notably different from previous findings on 2D materials with hexagonal or triangular moiré superlattices [22,47,49], where flat bands appear only at small twist angles and require larger unit cells, which leads to lower electron density. The bandwidth obtained in the present work is also smaller than that of twisted bilayer graphene and other common twisted bilayers of 2D materials. The corresponding charge density distribution for the flat band is plotted in Fig. 4B. The states are well localized at the center of the supercell and are dominated by the O *p_z_* orbitals, which is consistent with the narrow bandwidth.

Based on the findings discussed above, we propose the potential to design ultra-small polar topological structures and achieve their high-density integration through twisting engineering, going beyond the common mechanism of geometric confinement for polar topologies. Polar skyrmions emerge in the SrTiO_3_ moiré superlattice at large twisting angles of 36.87° and 22.62°, with each layer down to the monolayer limit, which is even thinner than the minimum thickness required to sustain ferroelectricity in ferroelectric PbTiO_3_ thin films (~3 unit cells) [13,50], indicating the absence of critical thickness restrictions for ferroelectricity and polar skyrmion in moiré superlattices. Although SrTiO_3_ is a nonferroelectric material, the polarization in the twisted SrTiO_3_ structure is on the same order as that in ferroelectric BaTiO_3_ and PbTiO_3_ due to the strong interlayer coupling effects. Thus, twisting engineering of perovskite oxides overcomes the critical size limitation for ferroelectricity and provides a novel mechanism to achieve atomic-scale topological structures with large polarizations. In addition to the ultrathin dimension, the in-plane modulation period of skyrmions can contribute to achieving record-breaking high-density data storage. Specifically, the moiré superlattices with twist angles of 36.87° and 22.62° host densities of 1.3 × 10^18^ m^−2^ and 5.1 × 10^17^ m^−2^ polar skyrmion array elements, with each skyrmion measuring approximately 0.87 and 1.40 nm in size, respectively. This contributes to a theoretical data storage density of up to 776.1 and 299.7 terabits per square inch theoretically, assuming that each skyrmion stores 1-bit data. To the best of our knowledge, the current miniaturization and high integration of polar skyrmions surpass all previous reports of domain density in the state-of-the-art ferroelectric structures. For instance, the vortex domain has been theoretically predicted to be stable at a minimum size of 3.2 nm in nanostructures [51], whereas the smallest period of polar skyrmions has been observed to be approximately 5 to 6 nm in the PbTiO_3_/SrTiO_3_ superlattice [52] and bilayer [53].

It is worth noting that twist engineering in bilayer structures causes nonlocal modulation of the resulting polar skyrmions, while the individual manipulation of polar configurations remains a challenge. A very recent study [42] proposed that polar vortices in twisted bilayer MoS_2_ can be locally modulated through subtle interlayer displacement and strain induced by the introduction of an electron-beam-mediated crack. This approach may also be applied to twisted bilayer SrTiO_3_ for the local modulation of polar skyrmions, as the polarization in SrTiO_3_​ is highly sensitive to strain. Moreover, the polar skyrmions in the twisted structure can be manipulated using a local electric field. To demonstrate this, we constructed a machine-learning potential to perform large-scale atomistic simulations (see the Supplementary Materials for details on the methodology). We apply a local vertical electric field of 10^3^ kV/cm to one moiré superlattice (the central square area) in the 5 × 5 × 1 unit cells of the moiré superlattice with *θ* = 36.87°. Figure S13 shows the corresponding polarization distribution under the local electric field, revealing the disappearance of the skyrmion at the center of the film, while the neighboring skyrmion remains intact. Thus, the skyrmions can also be manipulated individually by applying a local electric field using techniques such as atomic force microscopy (AFM) in practice. Note that the actual local modulation area in experiments depends on the size of the AFM tip. Tips with radii ranging from 5 to 10 nm are commonly used, while the smallest available tip currently has a radius of 2 nm [54]. Using this AFM tip, we can achieve local modulation of skyrmions within a 5 × 5 unit cell region of the moiré superlattice (4.35 nm × 4.35 nm) in experiments. With the advancement of tip-based and other related technologies, it will be possible to manipulate even smaller regions. Therefore, ultrahigh-density polar skyrmions, which overcome the critical size limitation, along with the novel mechanism proposed in this study, have important implications both from a fundamental science perspective and for practical applications in high-density information storage.

## Conclusion

In summary, a new strategy for moiré engineering of the perovskite oxide SrTiO_3_ has been proposed to create atomic-scale ferroelectricity characterized by exotic domain structures, although SrTiO_3_ is an intrinsically nonferroelectric material. The electric polarization is maintained in SrTiO_3_ materials with extremely thin monolayer thickness, breaking the critical size limitation for ferroelectricity in traditional perovskite oxides. The emergent ferroelectricity, characterized by a polar skyrmion lattice, originates from the interlayer bonding effect, which contributes to remarkable ion displacement. Furthermore, we investigate the twist angle-dependent bandwidth of the valence band maximum and find the formation of an ultraflat band at a large angle of 12.68°, with a bandwidth smaller than that of twisted bilayer graphene. Our findings not only overcome the detrimental critical thickness limitation of ferroelectricity in traditional perovskite oxides but also present opportunities for developing ultrahigh-density polar skyrmion-based memories.

## Data Availability

The data that support the findings of this study are available from the corresponding author upon reasonable request.

## Supplementary Materials

Supplementary 1 Notes S1 to S4 Tables S1 and S2 Figs. S1 to S18 References
