# Supplementary material for: Creating Ferroelectricity and Ultrahigh-Density Polar Skyrmion in Paraelectric Perovskite Oxide Monolayers by Moiré Engineering
Source: Research (Wash D C). 2025 Feb 17;8:0621. doi: 10.34133/research.0621 (PMC11830887; doi:10.34133/research.0621)
Supplement: Supplementary 1 — Notes S1 to S4 Tables S1 and S2 Figs. S1 to S18 References [file research.0621.f1.docx]

Supporting Information for

**Creating ferroelectricity and ultrahigh-density polar skyrmion in paraelectric perovskite oxide monolayers by moiré engineering**

Tao Xu^1*^, Qian Tao^2^, Jiafei Pang^1^, Jingtong Zhang^3^, Sheng Li^4^, Ri He^2*^, Jie Wang^3^, Takahiro Shimada^1*^

*^1^Department of Mechanical Engineering and Science, Kyoto University, Nishikyo-ku, Kyoto 615-8540, Japan*

*^2^Ningbo Institute of Materials Technology and Engineering,* *Chinese Academy of Sciences, Ningbo 315201, China*

*^3^Department of Engineering Mechanics, School of Aeronautics and Astronautics, Zhejiang University, Hangzhou 310027, China*

*^4^Department of Civil and Earth Resources Engineering, Kyoto University, Nishikyo-ku, Kyoto 615-8540, Japan*

*Corresponding authors:

[xu.tao.44a@st.kyoto-u.ac.jp](mailto:xu.tao.44a@st.kyoto-u.ac.jp);

[heri@nimte.ac.cn](mailto:heri@nimte.ac.cn);

shimada.takahiro.8u@kyoto-u.ac.jp

**Supplementary Note S1: Construction of commensurate SrTiO_3_ moiré superlattices**

For a twisted bilayer where the lattice constant remains identical in both the top and bottom layers, the formation of a moiré superlattice is only commensurate with periodic boundary conditions at specific twist angles denoted as θ. The unit vector of untwisted SrTiO_3_ can be defined as $\boldsymbol{a}$ = (*a*_0_, 0 ,0), $\boldsymbol{b}$ = (0, *b*_0_, 0), $\boldsymbol{c}$ = (0, 0, *c*_0_). Here, *a*_0_, *b*_0_ , *c*_0_ represent the lattice constant of SrTiO_3_ monolayer respectively and *a*_0_ = *b*_0_ = *c*_0_ for the cubic crystal of SrTiO_3_. To form a twisted bilayer of SrTiO₃, each layer is independently rotated. For proper alignment between the upper and lower layers, their lattice constants must be identical. Additionally, due to the constraints imposed by periodic boundary conditions, only certain twist angles, defined by pairs of integers (m, n), are feasible. Once the rotation is completed, the in-plane superlattice vectors of the top layer can be rigorously defined as follows:

$\boldsymbol{A}_{\boldsymbol{up}}\boldsymbol{=}m\boldsymbol{a+}n\boldsymbol{b}$ (1)

$\boldsymbol{B}_{\boldsymbol{up}}\boldsymbol{=-}n\boldsymbol{a+}m\boldsymbol{b}$ (2)

In this work, the integers m and n are defined as positive integers, indicating that the upper layer is rotated in a counterclockwise direction relative to the origin unit cell. Subsequently, the lower layer is rotated in the opposite direction. The in-plane superlattice vectors of the lower layer can be precisely defined as follows:

$\boldsymbol{A}_{\boldsymbol{down}}\boldsymbol{=}n\boldsymbol{a+}m\boldsymbol{b}$ (3)

$\boldsymbol{B}_{\boldsymbol{down}}\boldsymbol{=-}m\boldsymbol{a+}n\boldsymbol{b}$ (4)

The angle between the upper and lower layers is given by:

$\theta=\arccos\left( \frac{\boldsymbol{A}_{\boldsymbol{up}}*\boldsymbol{A}_{\boldsymbol{down}}}{\left| \boldsymbol{A}_{\boldsymbol{up}}*\boldsymbol{A}_{\boldsymbol{down}} \right|} \right)=arccos(\frac{2mn}{m^{2}+n^{2}})$ (5)

The total count of unit cells in the moiré superlattice and the corresponding moiré period λ are:

$N=8(m^{2}+n^{2})$ (6)

and

$\lambda= \frac{a_{0}}{\sqrt{1-\cos\theta}}$ (7)

The construction of twisting model is illustrated in Figure S14 in the following.

**Supplementary Note S2: Assessment of the stability of bilayer structures with different terminations**

The freestanding STO has been successfully synthesized down to a single unit cell in experiments, exhibiting excellent long-range crystallinity as confirmed by TEM observations [1]. For the construction of twisted bilayer, three terminations including the TiO_2_-TiO_2_ termination, SrO-SrO termination, and TiO_2_-SrO termination are considered. Their stabilities are assessed by calculated the formation energy of the slab $\Omega_{form}^{i}$ as following:

$$\Omega_{form}^{SrO-SrO}=\frac{1}{2S}[(E_{slab}^{SrO-SrO}-N_{Sr}E_{bulk}-{\mu_{O}(N}_{O}-3N_{Sr})-{\mu_{Ti}(N}_{Ti}-N_{Sr})]$$

(8)

$$\Omega_{form}^{{TiO}_{2}-{TiO}_{2}}=\frac{1}{2S}[(E_{slab}^{{TiO}_{2}-{TiO}_{2}}-N_{Ti}E_{bulk}-{\mu_{O}(N}_{O}-3N_{Ti})-{\mu_{Sr}(N}_{Sr}-N_{Ti})]$$

(9)

$\Omega_{form}^{SrO-{TiO}_{2}}=\frac{1}{2S}(E_{slab}^{SrO-{TiO}_{2}}-N_{Ti}E_{bulk})$ (10)

where $E_{slab}^{i}$ is the total energy of the slab modeling the *i* termination, $E_{\mathrm{bulk}}$ is the total energy of SrTiO_3_ bulk crystal,$N_{Sr}$, $N_{Sr}$ and $N_{Ti}$ is the number of Sr, Ti and O atoms contained in the slab, S is the surface unit area.$\mu_{Sr}$, $\mu_{Ti}$ and $\mu_{O}$ is the chemical potential of the Sr, Ti and O atomic species, respectively.

The chemical potential of SrTiO_3_ varies with the surrounding environment. The main reason is that the constitution of chemical potential of Sr, Ti, and O need to meet the thermodynamic equilibrium conditions. Additionally, the formation of secondary phases, such as SrO and TiO_2_, must be considered to prevent the development of other solid solutions.

1. The Sr, Ti, and O chemical potential in SrTiO_3_ μ_α_ should be lower than the energy (*E_α_*) of each elemental solid. The chemical potential should meet the following formulations:

$\Delta u_{Ti}\leq0, \Delta u_{Sr}\leq0$, ${\Delta\mu}_{O}\leq0$ (11)

where $\Delta u_{\alpha} = u_{\alpha}-E_{\alpha}$ is the difference in the μ_Sr_, μ_Ti_, and μ_O_ of the Sr, Ti, and O atom and the Sr, Ti, and O elemental solid, respectively. There are the following expressions: ${\Delta\mu}_{Sr}=\mu_{Sr}- E_{Sr}^{bulk}$, ${\Delta\mu}_{Ti}=\mu_{Ti}- E_{Ti}^{bulk}$, ${\Delta\mu}_{O}=\mu_{O}- E_{O2}^{mol}/2$, where the $E_{Sr}^{bulk}$, $E_{Ti}^{bulk}$ and $E_{O2}^{mol}/2$ are the energies of the Sr atom in the bulk cubic structure, of a Ti atom in hcp bulk metal, and the O atom in the O_2_ molecule in the gas phase by considering spin-polarization, respectively.${\Delta\mu}_{Sr}$, ${\Delta\mu}_{Ti}$ and ${\Delta\mu}_{O}$ are also confirmed by the following relationship

1. From the thermodynamic equilibrium condition, the value formation enthalpy of SrTiO_3_ need to equal to the sum values of ${\Delta\mu}_{Sr}$, ${\Delta\mu}_{Ti}$, and ${\Delta\mu}_{O}$ to ensure finally SrTiO_3_ bulk stability:

$\Delta H(SrTiO_{3})\equiv(u_{Ti}+u_{Sr}+3u_{O})-(E_{Ti}+E_{Sr}+3E_{O})= \Delta u_{Sr}+\Delta u_{Ti}+3\Delta u_{O}$ (12)

where the $\Delta H(SrTiO_{3})$ is the formation enthalpy of SrTiO_3_.

1. The Sr, Ti, and O chemical potentials need to keep lower than the formation enthalpy of SrO and TiO_2_ compounds:

$\Delta u_{Sr}+\Delta u_{O} \leq\Delta H(SrO)$ (13)

$\Delta u_{Ti}+2\Delta u_{O} \leq\Delta H(TiO_{2})$ (14)

$2\Delta u_{Ti}+3\Delta u_{O} \leq\Delta H({Ti}_{2}O_{3})$ (15)

$\Delta u_{Ti}+\Delta u_{O} \leq\Delta H(TiO)$ (16)

where $\Delta H(SrO)$, $\Delta H(TiO_{2})$, $\Delta H({Ti}_{2}O_{3})$, and $\Delta H(TiO)$ are the formation enthalpy of SrO, TiO_2_. Ti_2_O_3_, and TiO, respectively.

The ${\Delta\mu}_{Sr}$, ${\Delta\mu}_{Ti}$, and ${\Delta\mu}_{O}$ should be chosen form the range of chemical potentials that satisfy the above relationship. By meeting the stability of compounds and chemical potential, the phase diagram of tetragonal SrTiO_3_ is provided as the Figure S15. The upper and lower bounds of $\Delta u_{Sr}$ and $\Delta u_{Ti}$ are obtained, and then we calculate the formation energies for the TiO_2_-TiO_2_, SrO-SrO, and TiO_2_-SrO terminations at points A, B, C, D and E, respectively. The results are summarized in Table S2. From the data, we can infer that SrO-SrO termination exhibits the highest thermodynamic stability in the regions near points B, C, and D.

**Supplementary Note S3: Evaluation of local polarization**

The ferroelectric properties are evaluated through site-specific local polarization $p_{i}$, which interprets atomic displacements within a local unit cell *i* in relation to ferroelectricity and can be calculated by

$p_{i}=\frac{e}{\Omega_{c}} \omega_{j}Z_{j}u_{j}$ (17)

Here, $\Omega_{c}$ represents the volume of the local unit cell, *e* denotes the electron charge, $Z_{j}$ is the Born effective charge tensor [2, 3] and $u_{j}$ signifies the atomic displacement vector. The relaxed paraelectric thin film systems are used as the reference structure for determining displacement. The index *j* includes all atoms within the local unit cell. The local polarization is evaluated for each local unit cell, defined by the centered atom as illustrated in Figure S16. For the Sr-centered lattice, weights *w* are set to 1 for Sr, 1/8 for Ti, and 1/4 for O. For the Ti-centered lattice, weights *w* are 1/8 for Sr, 1 for Ti, and 1/2 for O. For O-centered lattice in the SrO or TiO2 plane, weights *w* are 1/4 for Sr, 1/2 for Ti, and 1 for centered O and 1/4 for other O. The detailed formulas for calculating local polarization in different atom-centered unit cells are as follows:

$\begin{matrix} \mathbf{P}_{Sr}=\frac{e}{\Omega_{c}}\sum_{j} w{}_{j}\mathbf{Z}_{j}^{*}\mathbf{u}_{j}=\frac{e}{\Omega_{c}}[\frac{1}{2}*\mathbf{Z}_{Sr}^{*}*\mathbf{u}_{Sr}+\frac{1}{8}*\mathbf{Z}_{Ti}^{*}*(\mathbf{u}_{Ti1}+\mathbf{u}_{Ti2}+\mathbf{u}_{Ti3}+\mathbf{u}_{Ti4}+\mathbf{u}_{Ti5}+\mathbf{u}_{Ti6}+\mathbf{u}_{Ti7}+\mathbf{u}_{Ti8}) \\ -\frac{1}{4}*\mathbf{Z}_{O\_type1}^{*}*(\mathbf{u}_{O9}+\mathbf{u}_{O10}+\mathbf{u}_{O11}+\mathbf{u}_{O12}) \\ -\frac{1}{4}*\mathbf{Z}_{O\_type2}^{*}*(\mathbf{u}_{O1}+\mathbf{u}_{O2}+\mathbf{u}_{O3}+\mathbf{u}_{O4}+\mathbf{u}_{O5}+\mathbf{u}_{O6}+\mathbf{u}_{O7}+\mathbf{u}_{O8})] \end{matrix}$(18)

$\begin{matrix} \mathbf{P}_{Ti}=\frac{e}{\Omega_{c}}\sum_{j} w{}_{j}\mathbf{Z}_{j}^{*}\mathbf{u}_{j}=\frac{e}{\Omega_{c}}[\frac{1}{8}*\mathbf{Z}_{Sr}^{*}*(\mathbf{u}_{Sr1}+\mathbf{u}_{Sr2}+\mathbf{u}_{Sr3}+\mathbf{u}_{Sr4}+\mathbf{u}_{Sr5}+\mathbf{u}_{Sr6}+\mathbf{u}_{Sr7}+\mathbf{u}_{Sr8})+\mathbf{Z}_{Ti}^{*}*\mathbf{u}_{Ti} \\ -\frac{1}{2}*\mathbf{Z}_{O\_type1}^{*}*(\mathbf{u}_{O1}+\mathbf{u}_{O2}) \\ -\frac{1}{2}*\mathbf{Z}_{O\_type2}^{*}*(\mathbf{u}_{O3}+\mathbf{u}_{O4}+\mathbf{u}_{O5}+\mathbf{u}_{O6})] \end{matrix}$(19)

$\begin{matrix} \mathbf{P}_{Sr^{\text{*}}}=\frac{e}{\Omega_{c}}\sum_{j} w{}_{j}\mathbf{Z}_{j}^{*}\mathbf{u}_{j}=\frac{e}{\Omega_{c}}[\frac{1}{2}*\mathbf{Z}_{Sr}^{*}*(\mathbf{u}_{Sr1}+\mathbf{u}_{Sr2})+\frac{1}{4}*\mathbf{Z}_{Ti}^{*}*(\mathbf{u}_{Ti1}+\mathbf{u}_{Ti2}+\mathbf{u}_{Ti3}+\mathbf{u}_{Ti4}) \\ -\frac{1}{4}*\mathbf{Z}_{O\_type1}^{*}*(\mathbf{u}_{O1}+\mathbf{u}_{O2}+\mathbf{u}_{O3}+\mathbf{u}_{O4}+\mathbf{u}_{O5}+\mathbf{u}_{O6}+\mathbf{u}_{O7}+\mathbf{u}_{O8}) \\ -\frac{1}{4}*\mathbf{Z}_{O\_type2}^{*}*(\mathbf{u}_{O9}+\mathbf{u}_{O10}+\mathbf{u}_{O11}+\mathbf{u}_{O12})] \end{matrix}$(20)

$\begin{matrix} \mathbf{P}_{O\_type1}=\frac{e}{\Omega_{c}}\sum_{j} w{}_{j}\mathbf{Z}_{j}^{*}\mathbf{u}_{j}=\frac{e}{\Omega_{c}}[\frac{1}{4}*\mathbf{Z}_{Sr}^{*}*(\mathbf{u}_{Sr1}+\mathbf{u}_{Sr2}+\mathbf{u}_{Sr3}+\mathbf{u}_{Sr4})+\frac{1}{2}*\mathbf{Z}_{Ti}^{*}*(\mathbf{u}_{Ti1}+\mathbf{u}_{Ti2}) \\ -\frac{1}{4}\text{*}\mathbf{Z}_{O\_type2}^{*}*(\mathbf{u}_{O1}+\mathbf{u}_{O2}+\mathbf{u}_{O3}+\mathbf{u}_{O4}+\mathbf{u}_{O5}+\mathbf{u}_{O6}+\mathbf{u}_{O7}+\mathbf{u}_{O8}) \\ -\mathbf{Z}_{O\_type1}^{*}*\mathbf{u}_{O9}] \end{matrix}$(21)

$\begin{matrix} \mathbf{P}_{O\_type2}=\frac{e}{\Omega_{c}}\sum_{j} w{}_{j}\mathbf{Z}_{j}^{*}\mathbf{u}_{j}=\frac{e}{\Omega_{c}}[\frac{1}{4}*\mathbf{Z}_{Sr}^{*}*(\mathbf{u}_{Sr1}+\mathbf{u}_{Sr2}+\mathbf{u}_{Sr3}+\mathbf{u}_{Sr4})+\frac{1}{2}*\mathbf{Z}_{Ti}^{*}*(\mathbf{u}_{Ti1}+\mathbf{u}_{Ti2}) \\ -\frac{1}{4}\text{*}\mathbf{Z}_{O\_type1}^{*}*(\mathbf{u}_{O1}+\mathbf{u}_{O2}+\mathbf{u}_{O3}+\mathbf{u}_{O4}) \\ -\frac{1}{4}\text{*}\mathbf{Z}_{O\_type2}^{*}*(\mathbf{u}_{O5}+\mathbf{u}_{O6}+\mathbf{u}_{O7}+\mathbf{u}_{O8}) \\ -\mathbf{Z}_{O\_type2}^{*}*\mathbf{u}_{O9}] \end{matrix}$(22)

Specifically, the interpretive diagram and detailed formula for the calculation of unit-cell-based local polarization in the moiré unit-cell with *θ* = 36.87^o^ is demonstrated in Figure S18.

**Supplementary Note S4: Deep potential molecular dynamics**

The machine-learning potential on the twisted bilayer SrTiO_3_ was developed based on Deep Potential method. In recent works, we have constructed the machine-learning potential models for bulk and and membrane SrTiO_3_ using a training dataset obtained from the DFT calculations [4, 5]. Herein, we further extend our training datasets of bilayer SrTiO_3_ freestanding systems with different twist angles (θ) by concurrent learning procedure. Thus, the training data cover all the equilibrium and nonequilibrium stacking states in configuration space. The final training dataset contained ~3000 randomly perturbed supercells. We perform the molecular statics calculation by LAMMPS code [6]. A 5×5×1 unit cells of the moiré superlattice with *θ* = 36.87^o^ was used to model the local electric field application. We introduced an external electric field (~10^3^ kV/cm) by applying an additional electric force (~10^-3^ eV/Å) in atomistic simulation. For more details of deep neural network hyper-parameter and training process, please refer to our original literatures [4, 5].

**Table S1.** Structural parameters in different moiré superlattices.

| (*m*, *n*) | *θ* | *λ* (Å) | Number of atoms |
| --- | --- | --- | --- |
| (1, 2) | 36.87° | 8.70 | 70 |
| (2, 3) | 22.62° | 14.02 | 182 |
| (6, 1) | 18.92° | 23.66 | 518 |
| (3, 4) | 16.26° | 19.45 | 350 |
| (4, 5) 12.68° 24.90 574 | | | |

**Table S2.** The values of $\Omega_{surf}^{i}$of SrO-SrO termination, TiO_2_-TiO_2_ termination, and TiO_2_-SrO termination for points A, B, C and D, respectively.

| termination | A(J/m^2^) | B(J/m^2^) | C(J/m^2^) | D(J/m^2^) | E(J/m^2^) |
| --- | --- | --- | --- | --- | --- |
| SrO-SrO | 4.0485 | 3.6962 | 2.6952 | 2.6952 | 4.0485 |
| TiO_2_-TiO_2_ | 4.0380 | 4.3904 | 5.3914 | 5.3914 | 4.0380 |
| SrO-TiO_2_ | 3.9046 | 3.9046 | 3.9046 | 3.9046 | 3.9046 |


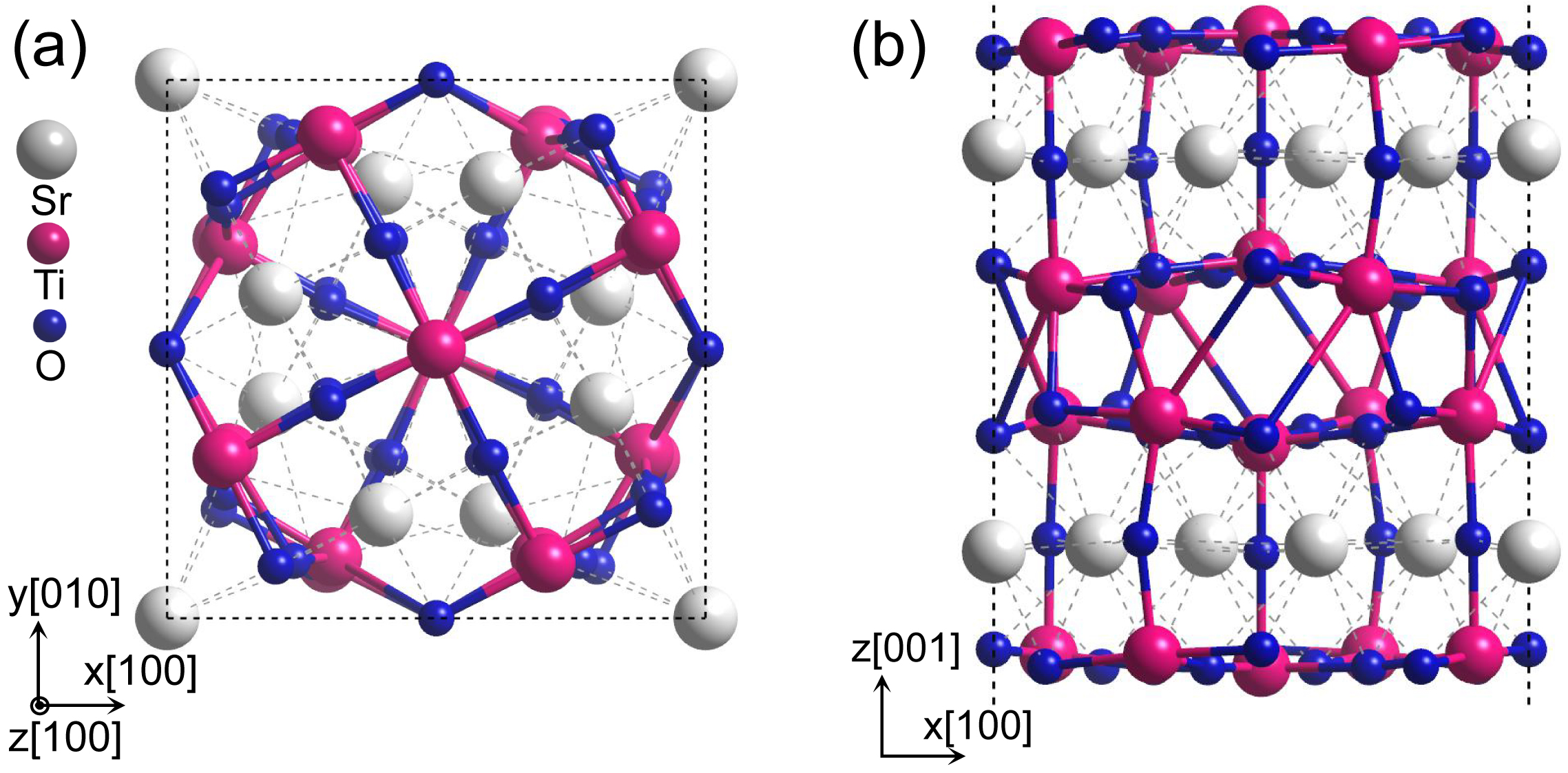


**Figure S1.** The structure of twisted SrTiO_3_ bilayer with TiO_2_-TiO_2_ termination. The rotation angle is 36.87^0^.


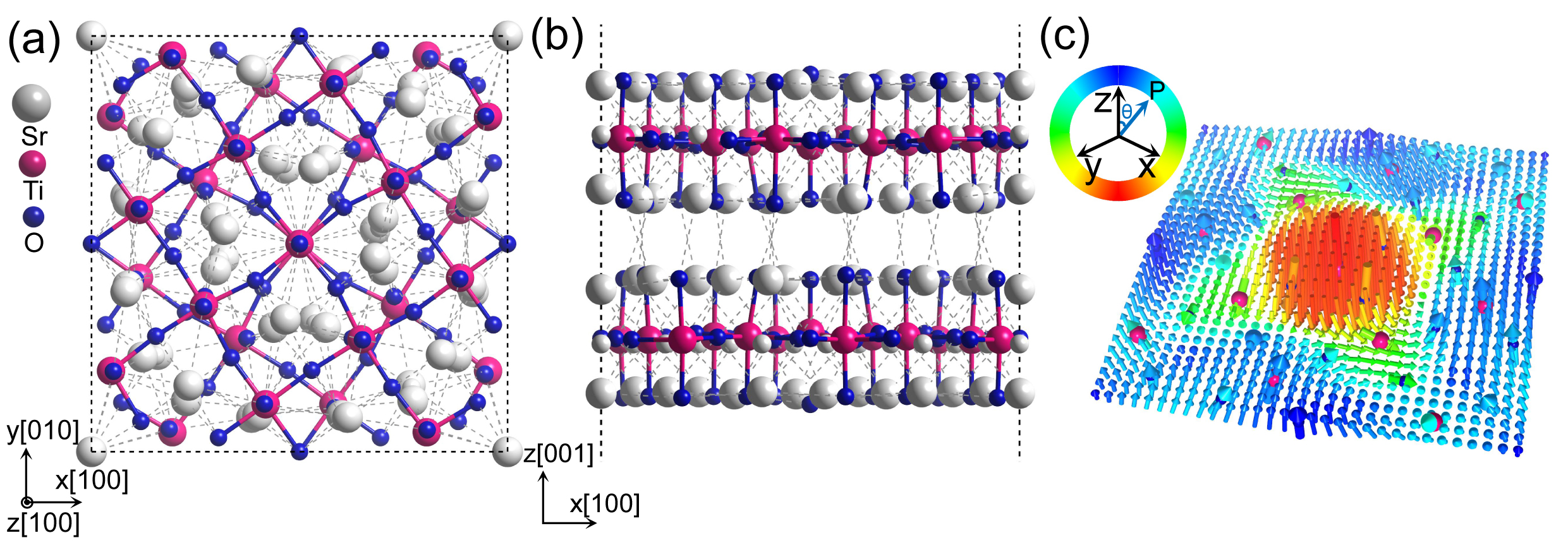


**Figure S2. (**a) In-plane and (b) out-of-plane projection of Moiré unit-cell with *θ* = 22.62^o^. (c) Polarization distribution and interpolated vector field of local polarization in the upper layer of moiré unit-cell with *θ* = 22.62^o^. Color denotes the angle from the z-direction.


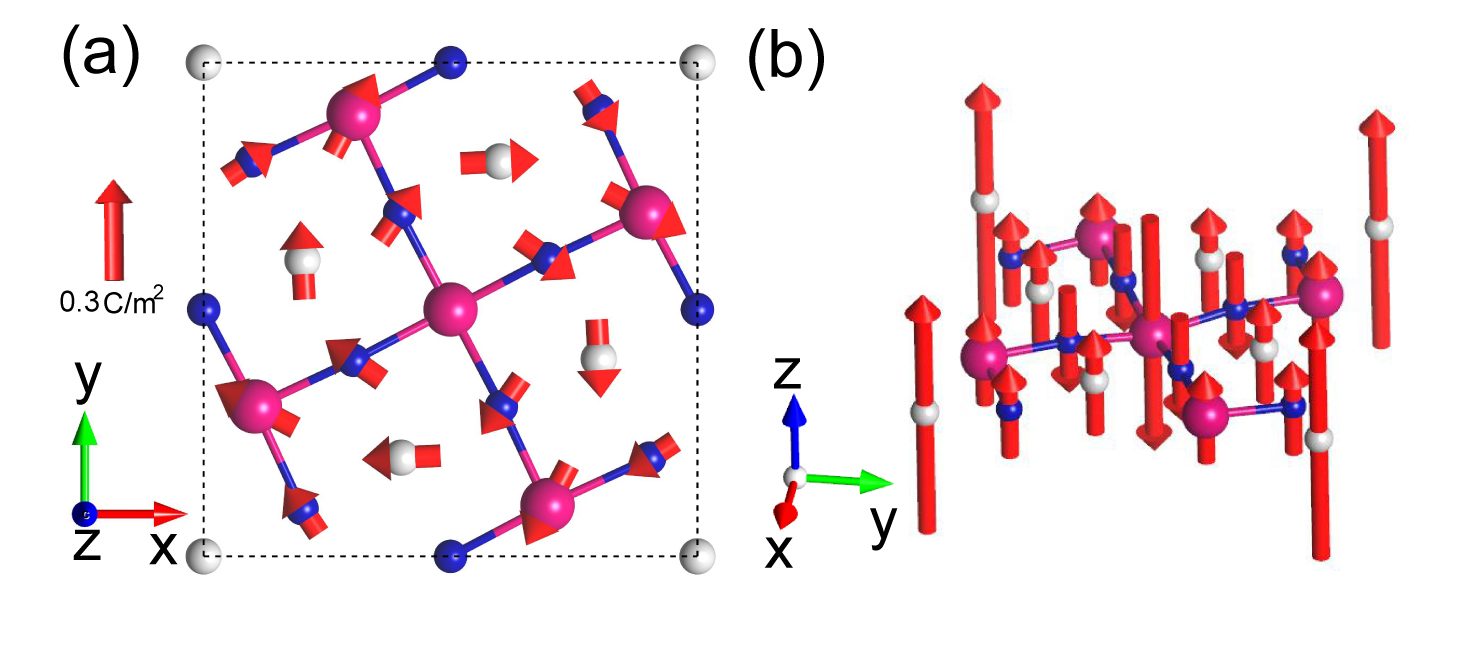


**Figure S3.** The in-plane and out-of-plane polarization projection of polarization in the upper layer of twisted SrTiO_3_ bilayer with *N* = 1 and *θ* = 36.8^o^.


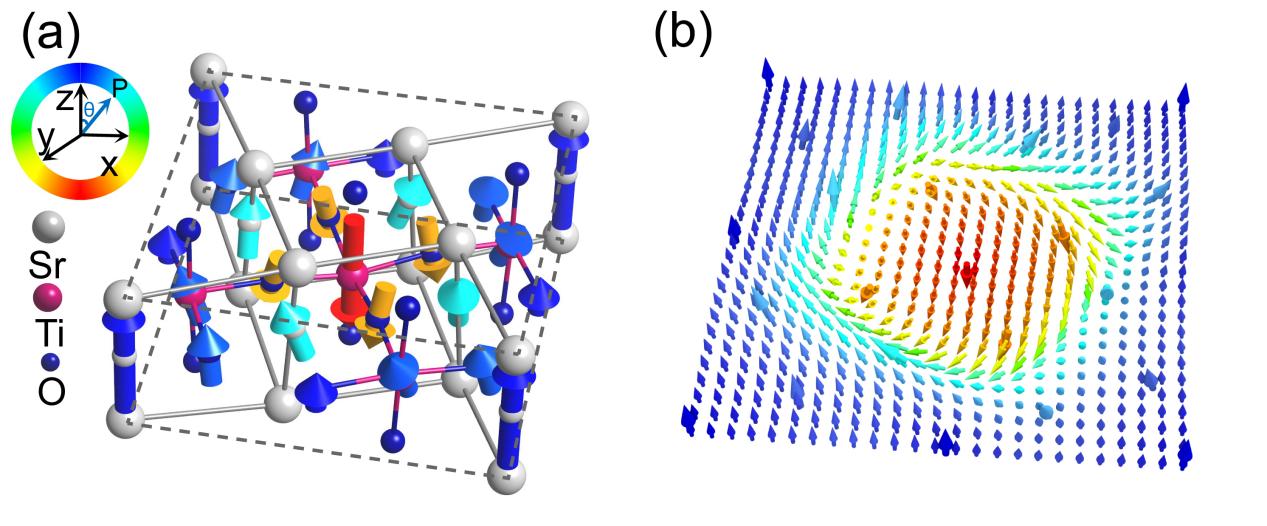


**Figure S4.** (a) Polarization distribution and (b) interpolated and normalized vector field of local polarization in the upper layer of SrTiO_3_ with θ = 36.87° based on PBESol pseudopotentials.


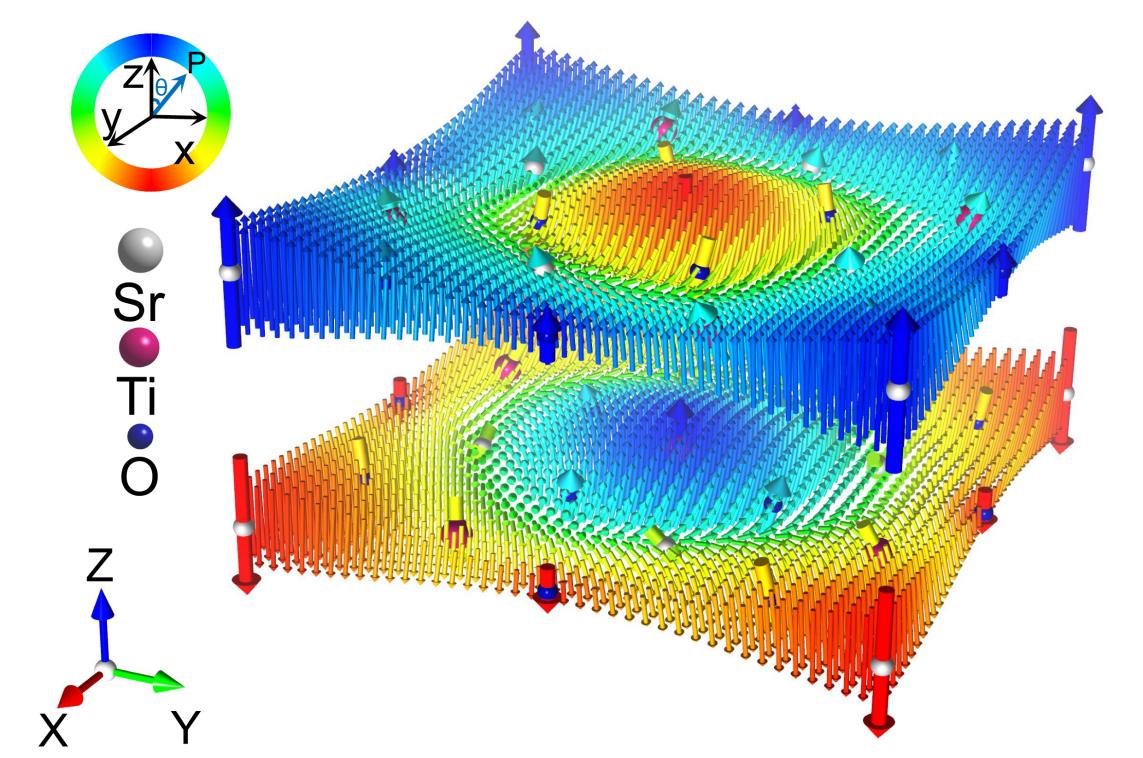


**Figure S5**. The distribution of local polarization in the twisted SrTiO_3_ bilayer with *N* = 1 and *θ* = 36.8^o^.


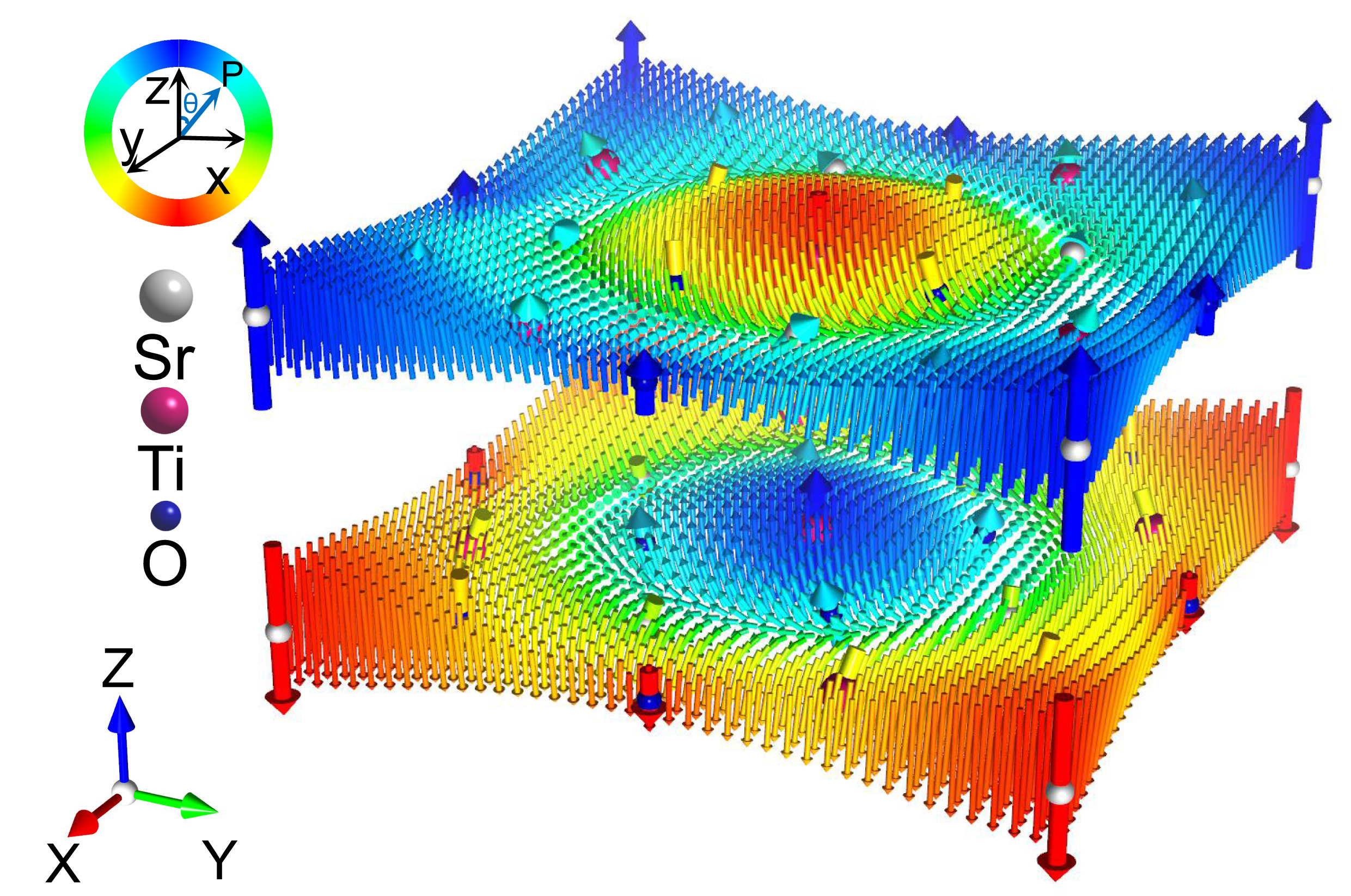


**Figure S6**. The distribution of local polarization in the twisted SrTiO_3_ bilayer with *N* = 1 and *θ* = –36.8^o^.


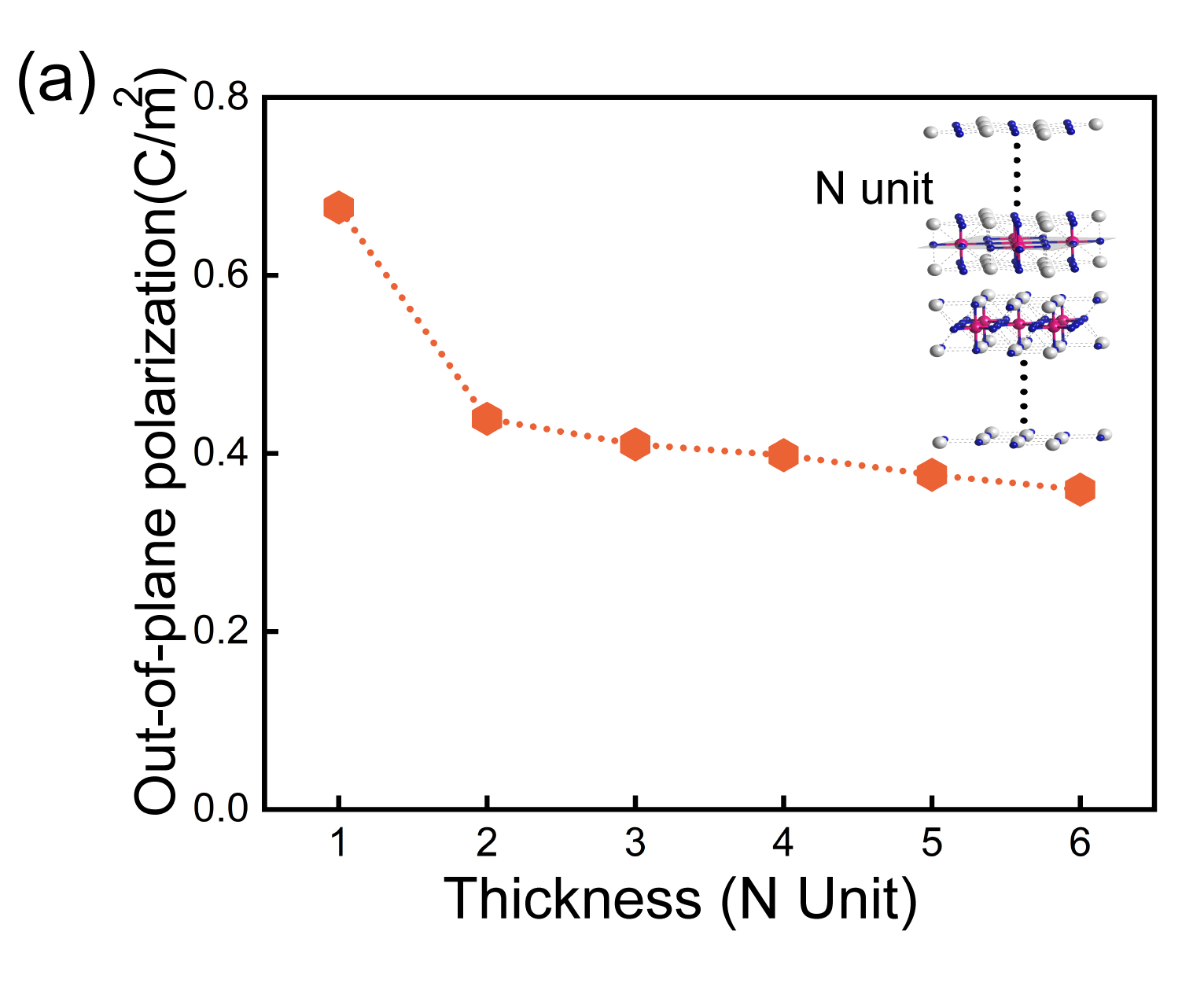


**Figure S7.** Thickness-dependent local polarization of the central unit cell in the interfacial sublayer of the twisted SrTiO₃ bilayer at a twist angle of *θ* = 36.8^o^.


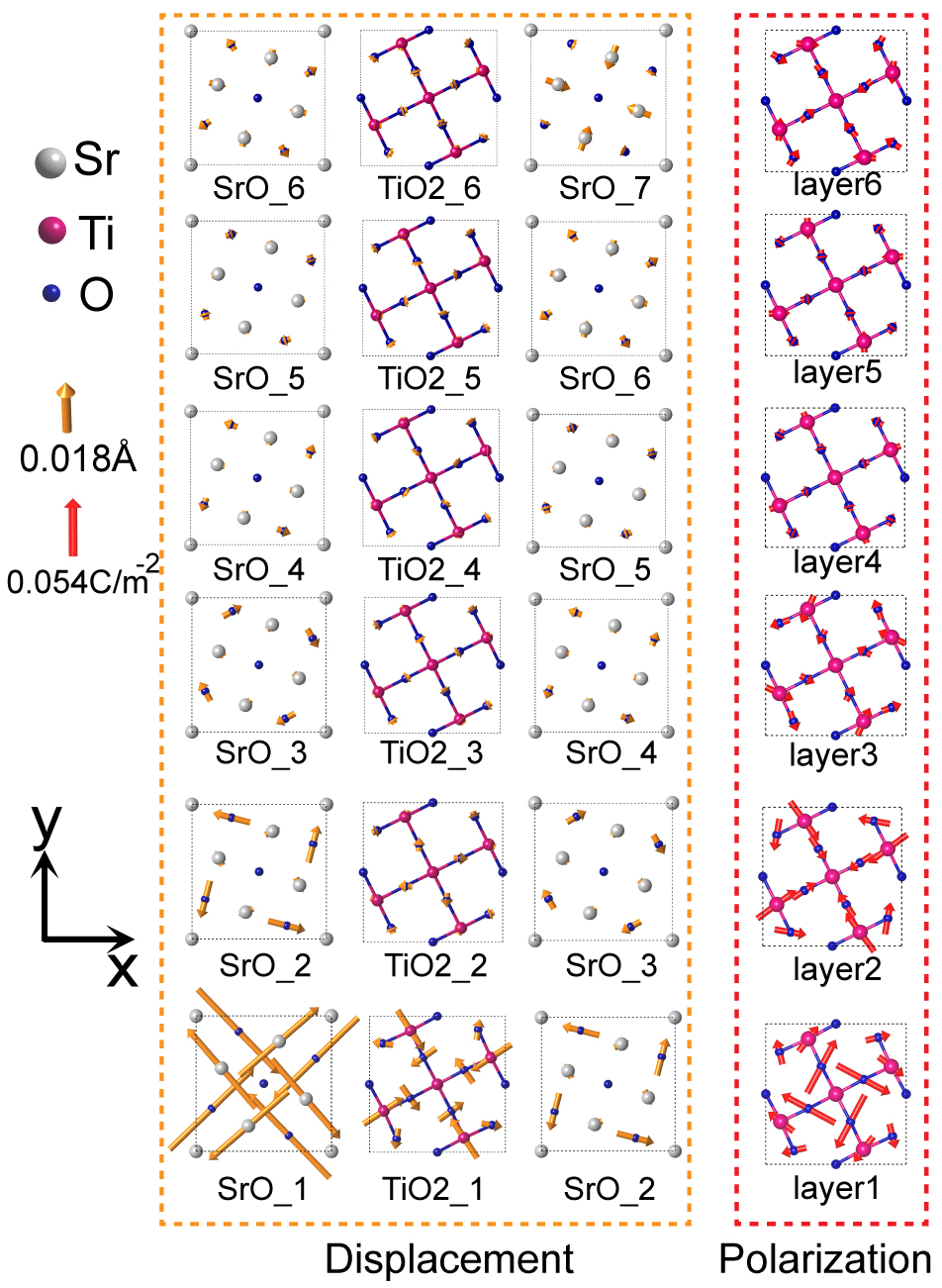


**Figure S8.** In-plane atomic displacement and local polarization in each unit cell of the upper layer of twisted SrTiO_3_ bilayer at *θ* = 36.87^o^ and *N* = 6. SrO_*n* and TiO2_*n* indicate the SrO and TiO2 atomic layer number from the interface to surface. The orange dashed box describes the different complex displacement fields between the layers, and the red dashed box shows the different in-plane polarizations patterns in each TiO_2_-centered unit cell of the upper layer from top to bottom for the model with *N* = 6.


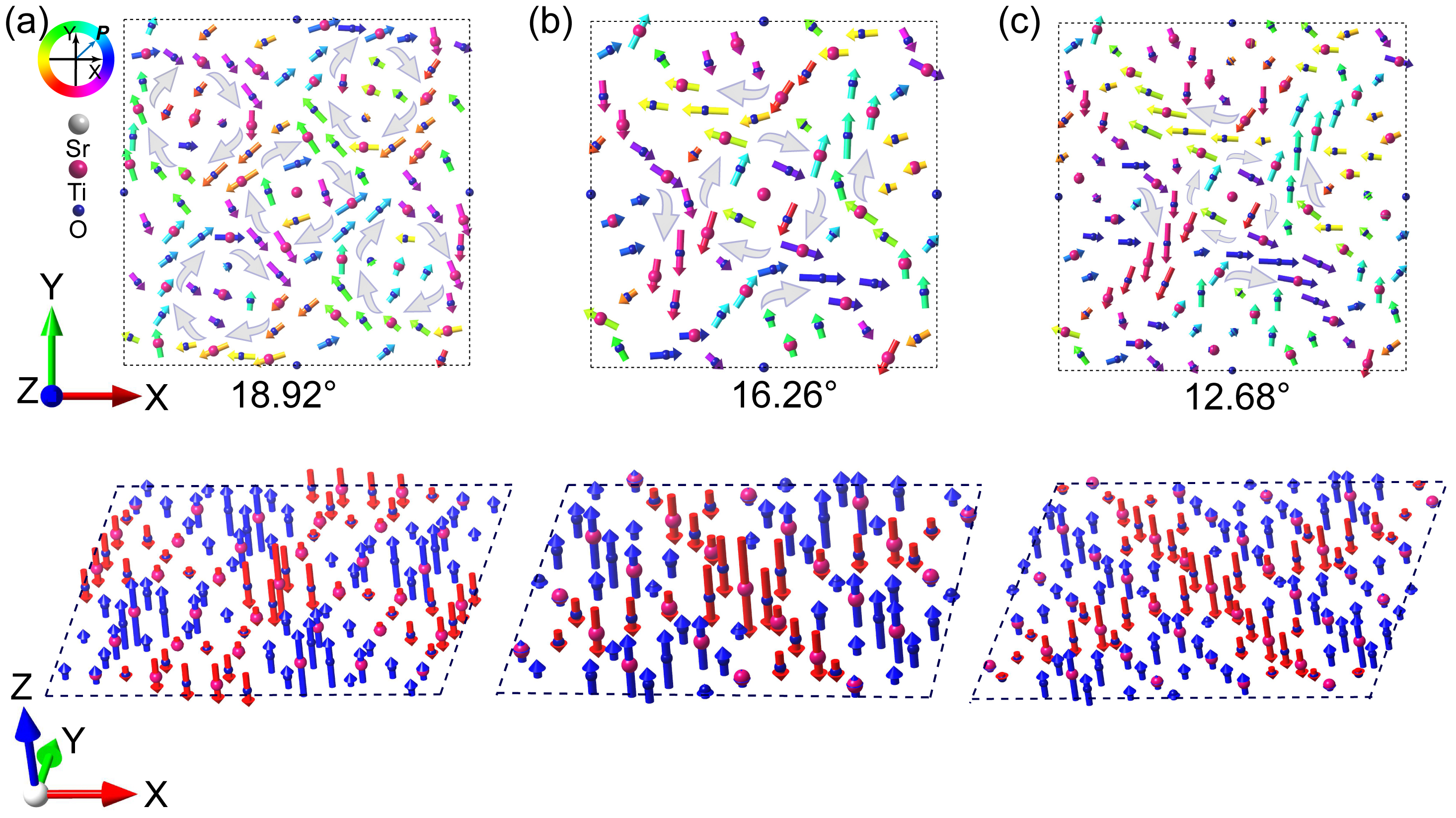


**Figure S9.** The in-plane (upper panels) and out-of-plane (lower panels) local polarization in the upper layer of moiré unit-cell for (a) *θ* = 18.92° (b) *θ* = 16.26° (c) *θ* = 12.68°. The red and blue arrows in the lower panels represent downward and upward local polarization, respectively.


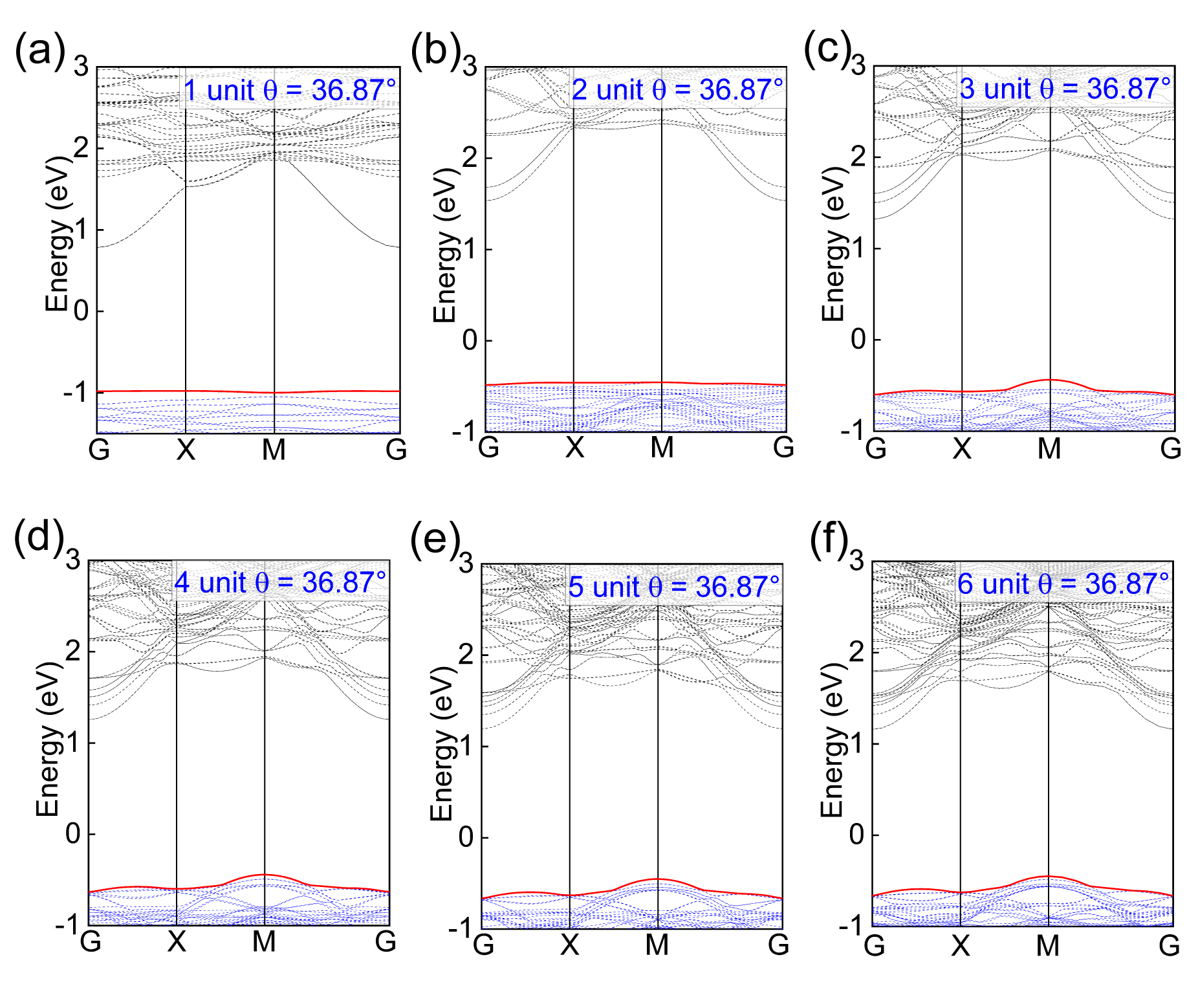


**Figure S10.** Band structures of twisted SrTiO_3_ bilayer at *θ* = 36.8^o^ with different thickness.


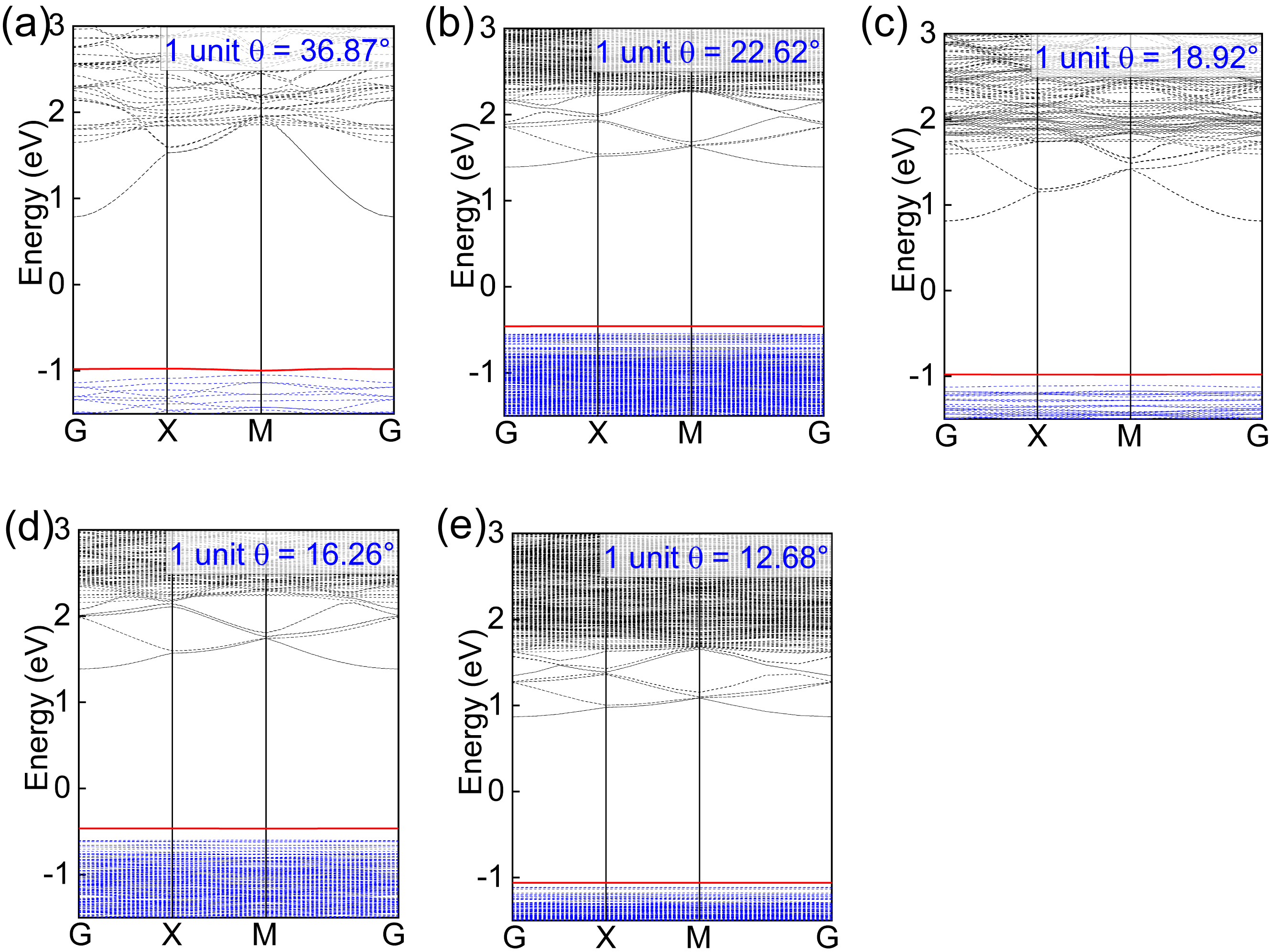


**Figure S11.** Band structures of twisted SrTiO_3_ bilayers at different twisting angles with *N* = 1.


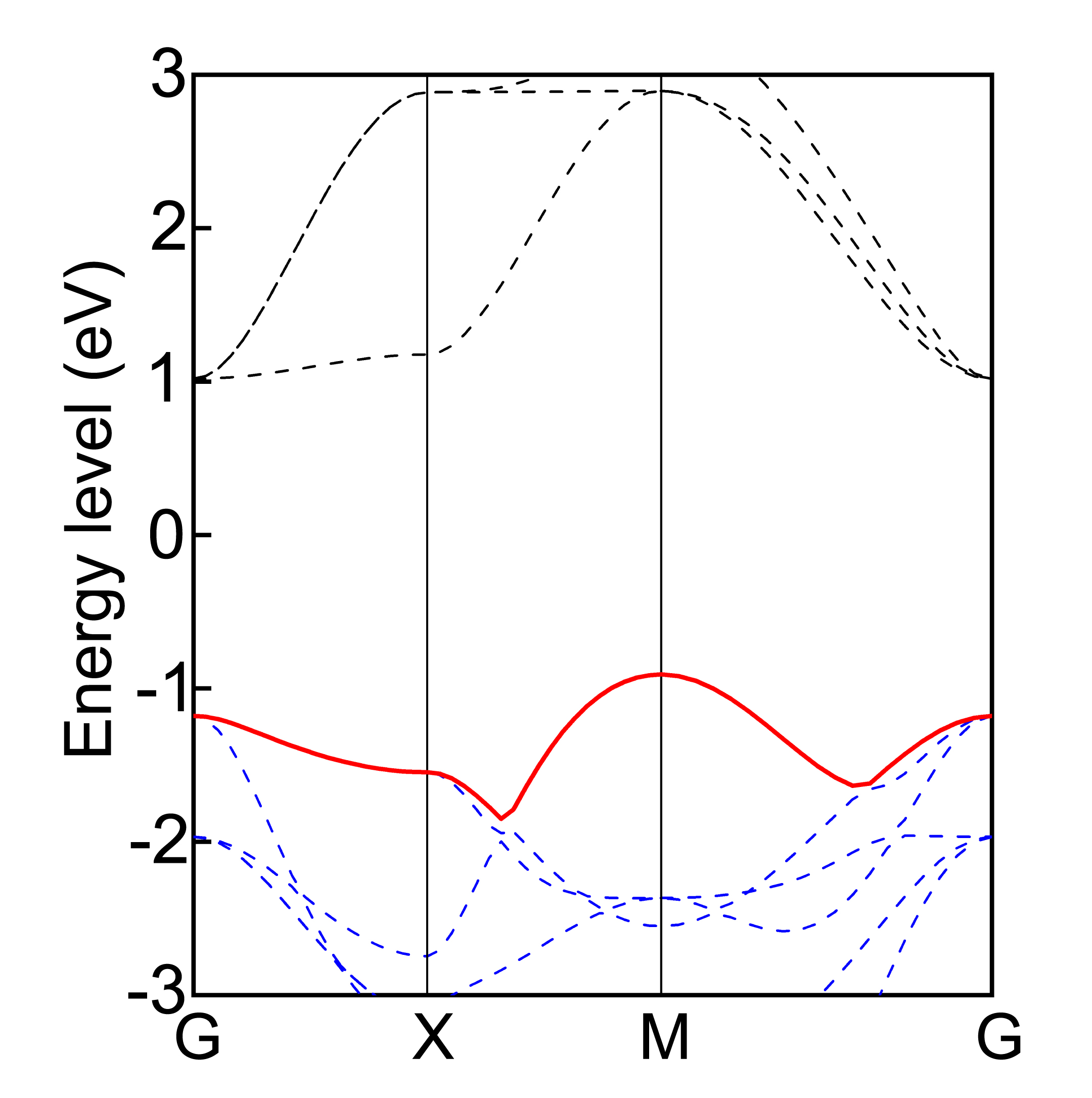


**Figure S12.** Band structure of bulk SrTiO_3_.


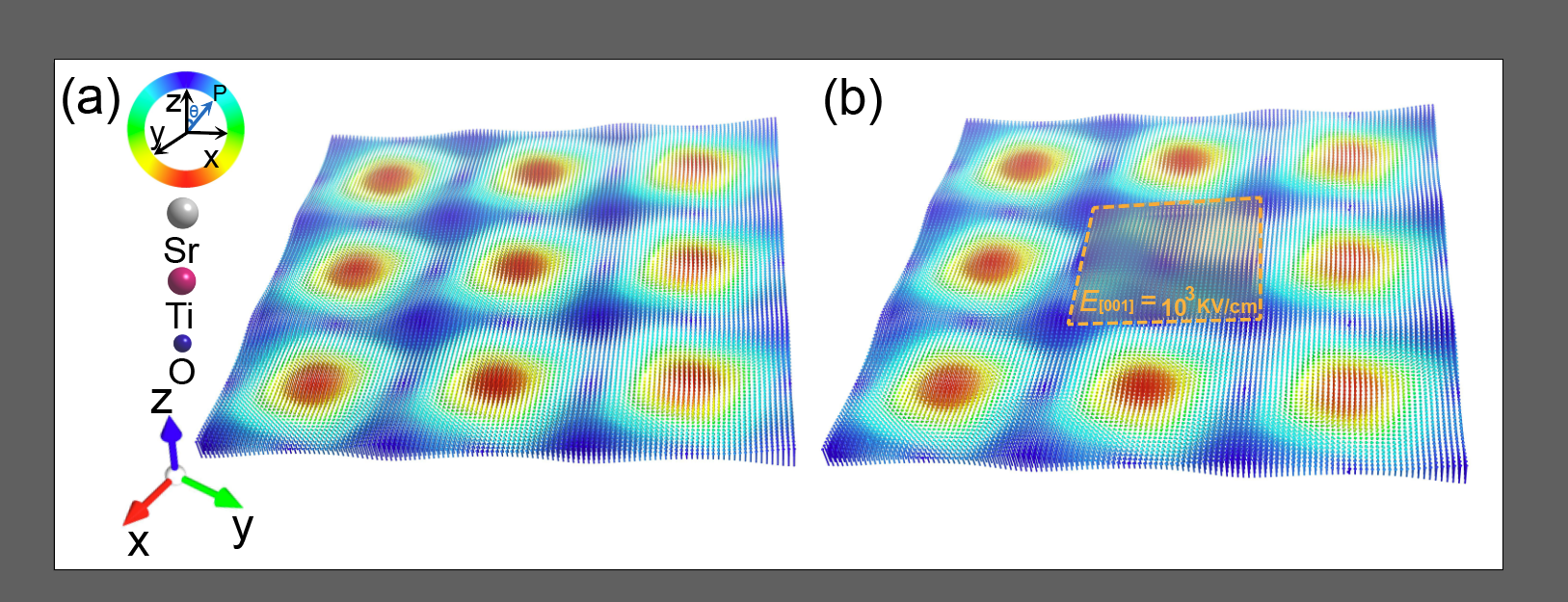


**Figure S13.** Polar configurations in the 5×5×1 unit cells of the moiré superlattice with *θ* = 36.87^o^ (a) before and (b) after the application of a local central electric field.


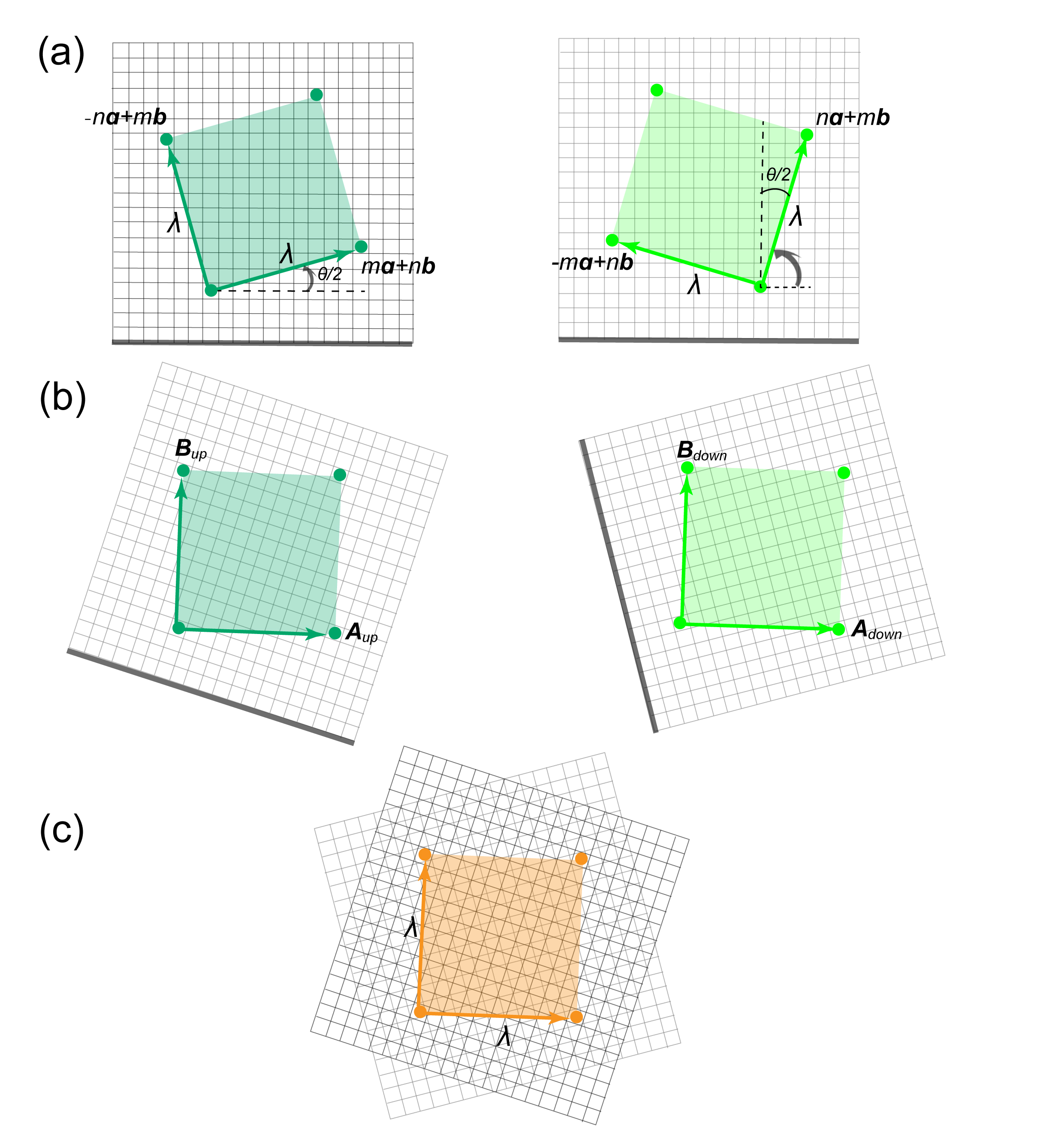


**Figure S14.** Construction of twisting model.


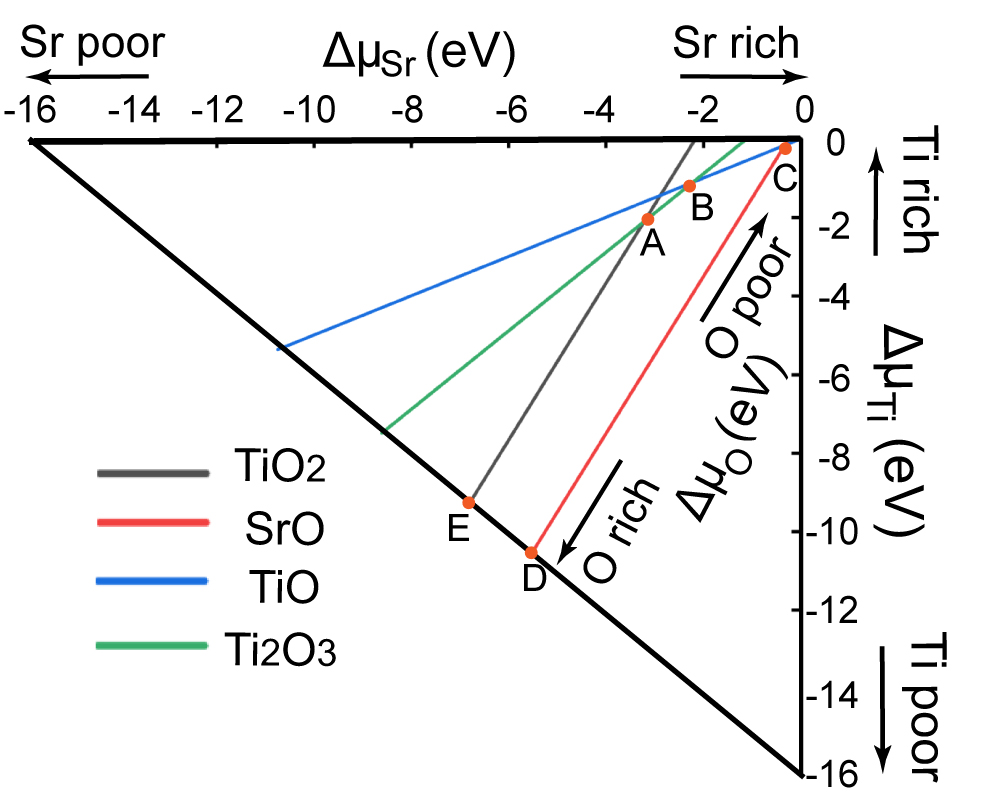


**Figure S15.** Phase diagram of cubic SrTiO_3_. The pentagonal area composed of A, B, C, D and E shows the chemical stability range of SrTiO_3_.


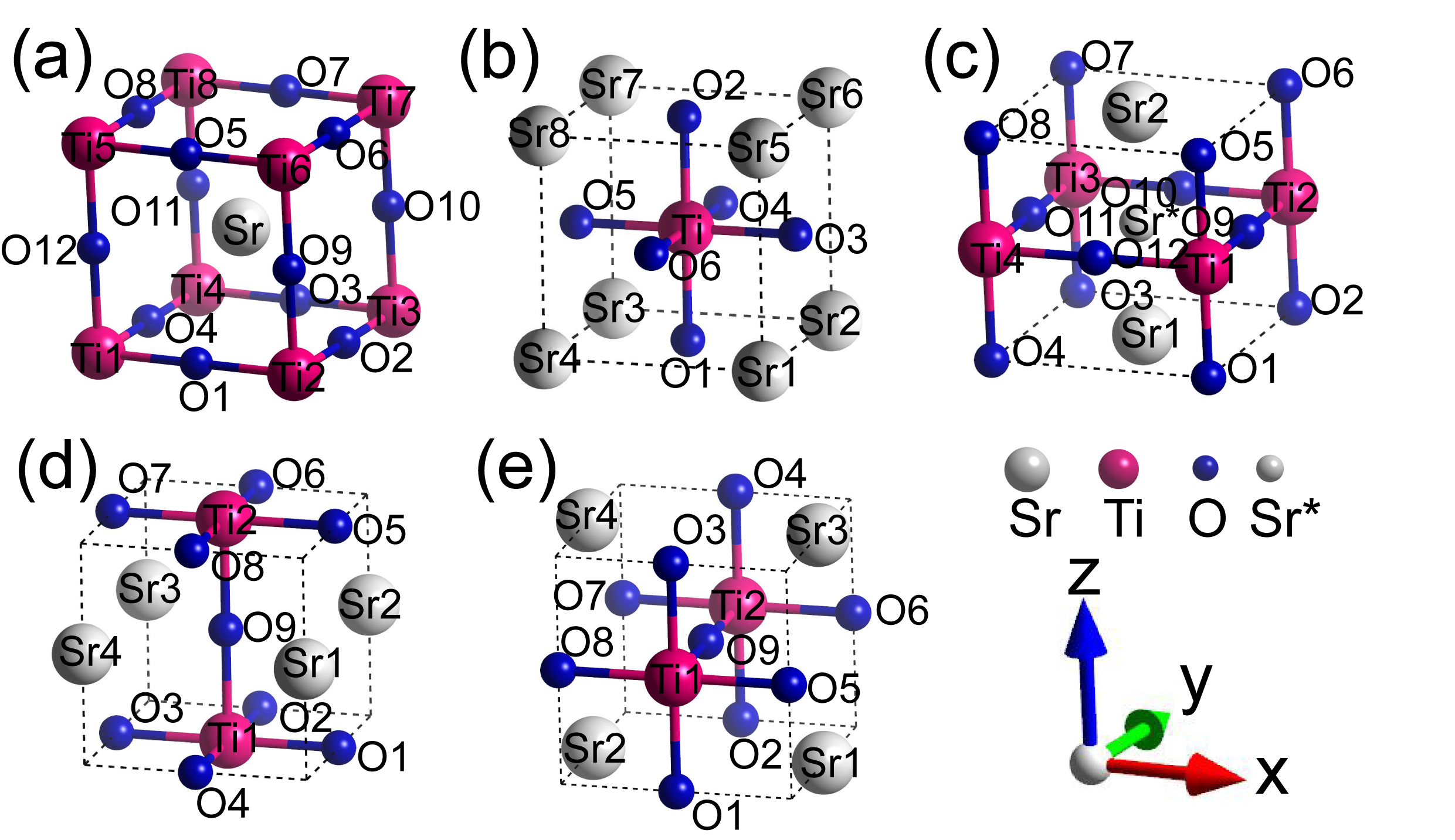


**Figure S16.** Local polarization $p_{i}$. (a) Sr-centered lattice. (b) Ti-centered lattice. (c) The Sr*-centered artificial unit cell, where Sr* represents the projected position of Sr onto the TiO₂ layer, has a local polarization that is equal to the average local polarization of the two adjacent SrO atomic layers. (d) O-centered lattice in the SrO plane. (e) O-centered lattice in the TiO2 plane.


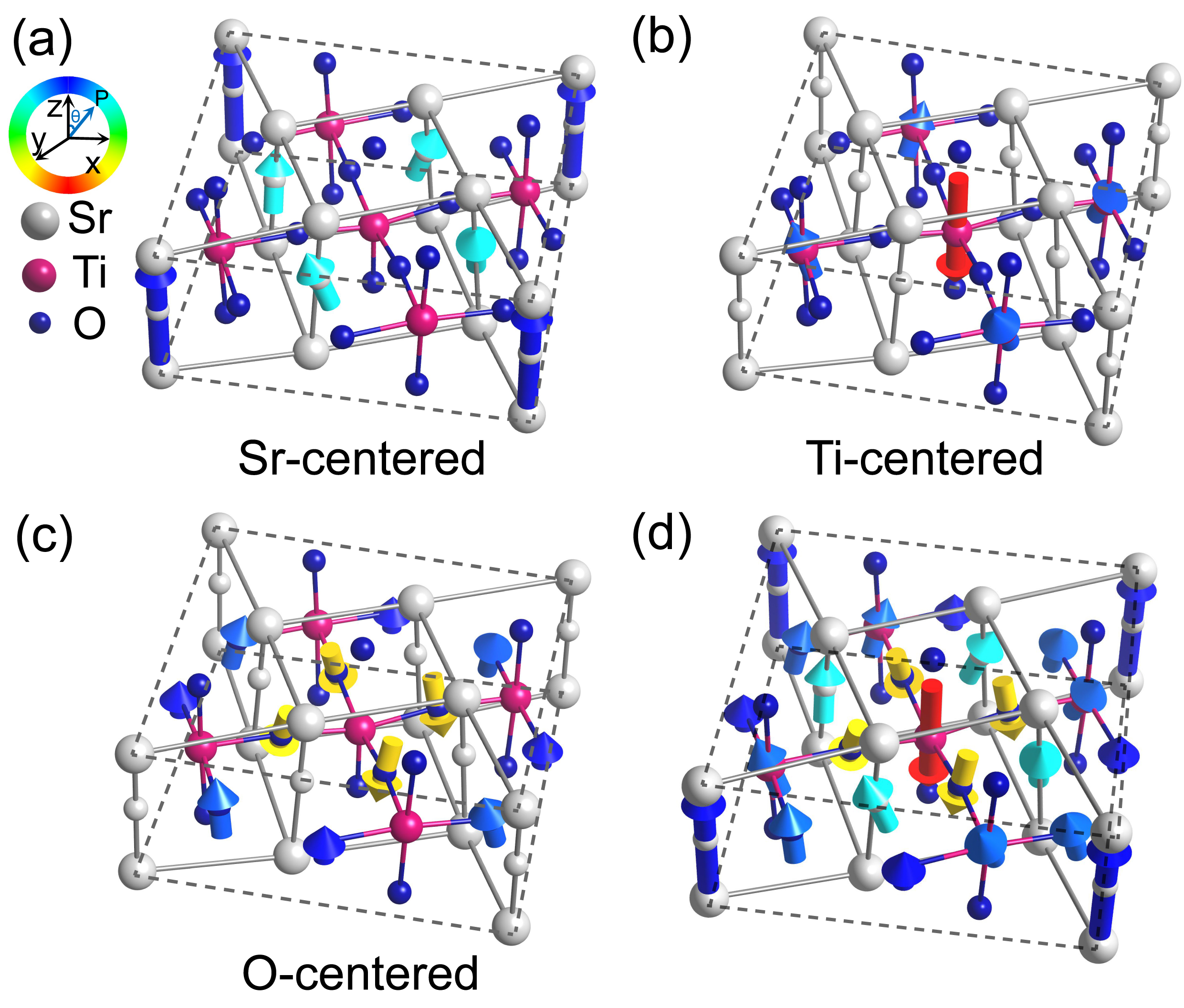


**Figure S17.** Polarization distribution in the upper layer of moiré unit-cell with *θ* = 36.87^o^, shown for different choices of unit cells: (a) Sr-centered, (b) Ti-centered, (c) O-centered, and (d) all atom-centered unit cells.


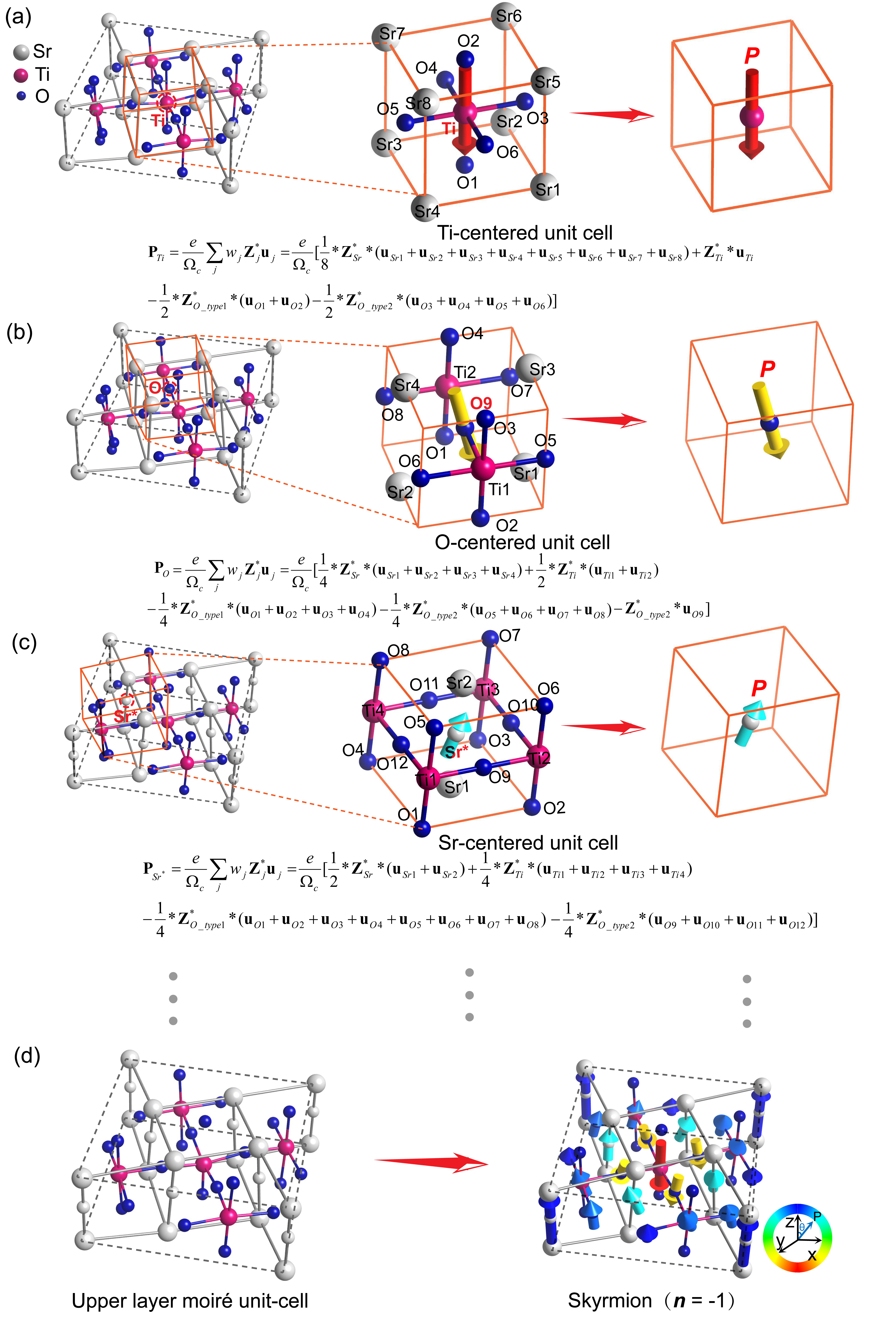


**Figure S18**. Interpretive diagram for the calculation of unit-cell-based local polarization in the moiré unit-cell with *θ* = 36.87^o^.

**Reference**

[1] D. M. Juraschek, P. Narang, Highly Confined Phonon Polaritons in Monolayers of Perovskite Oxides, Nano Lett. **21**, 5098 (2021).

[2] T. Xu, J. Wang, T. Shimada, and T. Kitamura, Direct approach for flexoelectricity from first-principles calculations: Cases for SrTiO_3_ and BaTiO_3_, J. Phys. Condens. Matter **25**, 415901 (2013).

[3] W. Zhong, R. D. King-Smith and D. Vanderbilt, Giant LO-TO Splittings in Perovskite Ferroelectrics, Phys. Rev. Lett. **72** 3618 (1994).

[4] R. He, H. Wu, L. Zhang, X. Wang, F. Fu, S. Liu, and Z. Zhong, Structural phase transitions in SrTiO_3_ from deep potential molecular dynamics, Phys. Rev. B **105**, 064104 (2022).

[5] R. He, H. Xu, P. Yang, K. Chang, H. Wang, and Z. Zhong, Ferroelastic Twin-Wall-Mediated Ferroelectriclike Behavior and Bulk Photovoltaic Effect in SrTiO_3_, [Phys. Rev. Lett. **132**, 176801 (2024).](https://doi.org/10.1103/physrevlett.121.026402)

[6] S. Plimpton, Fast Parallel Algorithms for Short-Range Molecular Dynamics, J. Comput. Phys. 117, 1 (1995).
